# Author Correction: How social relationships shape moral wrongness judgments

**DOI:** 10.1038/s41467-021-26493-4

**Published:** 2021-10-26

**Authors:** Brian D. Earp, Killian L. McLoughlin, Joshua T. Monrad, Margaret S. Clark, Molly J. Crockett

**Affiliations:** Department of Psychology, Yale University, New Haven, CT USA

**Keywords:** Human behaviour, Psychology

Correction to: *Nature Communications* 10.1038/s41467-021-26067-4, published online 1 October 2021.

The original version of this Article contained an error in Figure 4, in which the column labels “Reciprocity” and “Mating” were inadvertently placed in the wrong order. The correct order is “Mating” followed by “Reciprocity”. This has been corrected in both the PDF and HTML versions of the Article.

