# Author Correction: How social relationships shape moral wrongness judgments

**DOI:** 10.1038/s41467-024-52274-w

**Published:** 2024-09-10

**Authors:** Brian D. Earp, Killian L. McLoughlin, Joshua T. Monrad, Margaret S. Clark, Molly J. Crockett

**Affiliations:** Department of Psychology, Yale University, New Haven, CT USA

Correction to: *Nature Communications* 10.1038/s41467-021-26067-4, published online 01 October 2021

The original Article contained an error in the text in the results section titled ‘Relational norm profiles predict relationship-specific moral wrongness judgments out of sample’, where the term “on-target” was used incorrectly.

The original incorrect sentence read ‘Breaking the model down further, we find that target specificity was positively correlated with moral wrongness judgments (*p* < 0.001, 95% CI [0.34, 0.40]), indicating that the more “on-target” the effect of an action in weakening a given function, the more harshly that action was judged.’

The correct sentence reads ‘Breaking the model down further, we find that target specificity was positively correlated with moral wrongness judgments (p <.001, 95% CI [0.34, 0.40]), indicating that the more “off-target” the effect of an action (i.e., in weakening multiple functions) the more harshly that action was judged.’ This has been corrected in the PDF and HTML versions of the Article.

In addition there were a number of reporting errors in the original Article, none of which alter the conclusions. These errors arose inadvertently during the revision process. These errors in the Article and Supplementary Information are listed below.

Errors in the main text have been corrected in the PDF and HTML versions of the Article. The HTML has been updated to include a corrected version of the Supplementary Information. A script that outputs all of the results in the order of reporting in the manuscript can be found as Supplementary Information attached with this correction. The locations and wording from each of the changes are outlined in the marked up Manuscript and Supplementary Information file attached to this correction.

1. In the results section titled ‘Relational norms vary across common dyadic relationships’ in comparing the average prescription for the mating function against the scale midpoint, the *t-*value and *d-*value were misreported.

The original incorrect sentence reads **‘**Meanwhile, the mating function was negatively prescribed (i.e., proscribed) for most dyads (*M* across dyads = −63.02, *SD* = 62.01; lower than the scale midpoint with the same correction, *t*(8,459) = 230.76, *p* < 0.001, *d* = 1.82)…’

The correct sentence reads ‘Meanwhile, the mating function was negatively prescribed (i.e., proscribed) for most dyads (*M* across dyads = −63.02, *SD* = 62.01; lower than the scale midpoint with the same correction, *t*(8,459) = 93.48, *p* < 0.001, *d* = 1.02)…’

2. In the results section titled ‘Relational norms vary across common dyadic relationships’ gender differences in functional expectations for care and mating were reported, based on the results of an exploratory mixed linear effects regression model. The reported gender differences are correct. However participant age was not included in the final model as a fixed effect due to missing data for this variable.

The original incorrect sentence reads ‘After scaling the raw scores to each participant’s mean rating, we built a mixed linear effects regression model controlling for relevant demographic information (age, income, religiosity, and political orientation entered as fixed effects)…’

The correct sentence reads ‘After scaling the raw scores to each participant’s mean rating, we built a mixed linear effects regression model controlling for relevant demographic information (income, religiosity, and political orientation entered as fixed effects)…’

3. In the results section titled ‘Relational norms vary across common dyadic relationships’, the gender differences in mating expectations (by relationship) were misreported at the second decimal place for the close friend, colleague/classmate, and stranger relationships.

The original incorrect sentence reads ‘This divergence was most apparent for the friends-with-benefits (Mm − Mf = 0.27), roommate/housemate (Mm − Mf = 0.25), acquaintance (Mmale − Mfemale = 0.24), close friend (Mm − Mf = 0.24), colleague/classmate (Mm − Mf = 0.21), stranger (Mm − Mf = 0.17), and neighbor (Mm − Mf = 0.17) relationships.’

The correct sentence reads ‘This divergence was most apparent for the friends-with-benefits (Mm − Mfl = 0.27), roommate/housemate (Mm − Mf = 0.25), acquaintance (Mmale − Mfemale = 0.24), close friend (Mm − Mf = 0.25), colleague/classmate (Mm − Mf = 0.2), stranger (Mm − Mf = 0.16) and neighbor (Mm − Mf = 0.17) relationships.’

4. In the figure legend for Figure 2, the number of individual data points was incorrectly reported. The original incorrect sentence states ‘The raw data (*n* = 423 independent ratings per function per relationship; total *n* = 8460) are shown in individual dots; error bars represent the mean (dot) and + /− 1 SD (caps).’

The correct sentence reads ‘The raw data (*n* = 423 independent ratings per function per relationship; total *n* = 16,920) are shown in individual dots; error bars represent the mean (dot) and + /− 1 SD (caps).’

5. In the results section titled ‘Relational norm profiles predict relationship-specific moral wrongness judgments out of sample’ the overall Spearman’s correlation in K-S distances between dyads in relational norm space and moral judgment space as *r* = 0.43 (*p* = 0.003) was correctly reported. For the separate, function-by-function correlations, the correct values were reported in the Supplementary Information, Section 2.5.3, but not in the main manuscript.

The original incorrect sentence reads ‘Looking at the same K-S distances, but on a function-by-function basis (see Supplement Section 2.5.3. for the corresponding scatterplots), we find that the positive correlation between relational norm and moral judgment K-S distances holds for care (*r* = 0.48, *p* < 0.001), mating (*r* = 0.73, *p* < 0.001), and hierarchy (*r* = 0.31, *p* = 0.041), but not for reciprocity (*r* = −0.13, *p* = 0.39).’

The correct sentence reads ‘Looking at the same K-S distances, but on a function-by-function basis (see Supplement Section 2.5.3. for the corresponding scatterplots), we find that the positive correlation between relational norm and moral judgment K-S distances holds for care (*r* = 0.50, *p* < 0.001), mating (*r* = 0.69, *p* < 0.001), and hierarchy (*r* = 0.29, *p* = 0.05), but not for reciprocity (*r* = −0.10, *p* = 0.49).’

6. In the results section titled ‘Relational norms explain more variance in moral wrongness judgments than alternative models’ the AIC score for the relational norms model and the AIC score for the interdependence model were incorrectly reported.

The original incorrect sentence reads ‘In addition, measures of model fit suggest that the relational norms model (marginal *R*^2^ = 0.69, AIC = 841) performed substantially better than any of the alternative models: social closeness (marginal *R*^2^ = 0.44, AIC = 908.04), interdependence (marginal *R*^2^ = 0.44, AIC = 908.00), and genetic relatedness (marginal *R*^2^ = 0.44, AIC = 910.33).’

The correct sentence reads ‘In addition, measures of model fit suggest that the relational norms model (marginal *R*^2^ = 0.69, AIC = 841.36) performed substantially better than any of the alternative models: social closeness (marginal *R*^2^ = 0.44, AIC = 908.04), interdependence (marginal *R*^2^ = 0.44, AIC = 908.30), and genetic relatedness (marginal *R*^2^ = 0.44, AIC = 910.33).’

7. In Supplementary Table 2, Demographic information for Sample 1, one missing participant was omitted from the Race and Gender columns, respectively.

The corrected table lists these as additional items in the Race and N (%) columns ‘Missing’, ‘1 (0.24%)’ and additional items in the Gender and N (%) columns ‘Missing’, ‘1 (0.24%).’

8. In Supplementary Table 5, listing the most to least functionally polarized relationships, the standard deviation (SD) column was incorrectly calculated.

The original incorrect SD values read, in descending order ‘74.86, 74.10, 73.99, 70.32, 69.79, 65.45, 65.00, 64.30, 63.72, 61.21, 59.18, 55.39, 53.16, 50.93, 50.71, 46.35, 45.80, 39.87, 34.85, 33.46’.

The correct SD values read, in descending order ‘85.33, 85.20, 84.64, 80.92, 80.41, 74.32, 73.90, 73.87, 69.28, 67.51, 65.29, 60.77, 58.42, 57.44, 56.32, 52.77, 52.63, 42.21, 40.21, 37.93’.

9. In Supplementary Tables 7a–e and Supplementary Tables 11a–e, the conditional *R*^2^ values were miscalculated.

The original incorrect conditional *R*^2^ values in Supplementary Table 7a–e read **‘**.53’, ‘.72’,’ .62’, ‘.37’, ‘.46’. The correct conditional *R*^2^ values for Supplementary Table 7a–e read ‘0.0003’, ‘0.8’, ‘0.8’, ‘0.8’, ‘0.8.’

The original incorrect conditional *R*^2^ values in Supplementary Table 11a–e read ‘.56’, ‘.44’, ‘.42’, ‘.06’, ‘.58’. The correct conditional *R*^2^ values for Supplementary Table 11a–e ead ‘0.07, 0.59, 0.41, 0.07, 0.61’.

10. In Supplementary Table 7b, Mating, the upper confidence interval for Religiosity was incorrectly rounded to −.12, instead of-.11.

The original confidence interval for Religiosity reads ‘[−5.88, −.12]’. The correct confidence interval for Religiosity reads ‘[−5.88, −.11]’

11. In Section 2.2. of the Supplementary Information, two participants had missing data for Race, and one participant had missing data for Gender and Age; these participants were included in the total number of participants reported in the main manuscript, but were inadvertently left out of the demographic breakdown in Supplementary Table 10, Demographics of Sample 2 participants. Including these participants brings the number of female participants from 553 to 554, and the number of male participants from 758 to 759, with 1 missing (and 6 non-binary as reported); (*M*_*age*_ = 35.33, *SD*_*age*_ = 10.58). It also slightly changes associated percentages. Finally, a participant who had listed their age as 35.4751645 was originally not represented as being in the 28–37 age group due to a coding error; this has been corrected.

The original incorrect version of Supplementary Table 1 is:

**Table:** 

Age	*N* (%)	Race	*N* (%)	Gender	*N* (%)
18–27	329 (24.96%)	White	929 (70.49%)	Female	553 (41.96%)
28–37	561 (42.56%)	Black/African-American	175 (13.28%)	Male	758 (57.51%)
38–47	230 (17.45%)	Asian	92 (6.98%)	Other/Non-binary	6 (0.46%)
48–57	135 (10.24%)	Hispanic/Latinx	88 (6.68%)		
58+	63 (4.78%)	Other	19 (1.44%)		
Missing	0 (0.00%)	American Indian/Alaska Native	12 (0.91%)		
		Hawaiian/Pacific Islander	1 (0.08%)		

The correct version of Supplementary Table 10 is:

**Table:** 

Age	*N* (%)	Race	*N* (%)	Gender	*N* (%)
18–27	329 (24.92%)	White	930 (70.45%)	Female	554 (41.97%)
28–37	562 (42.58%)	Black/African-American	175 (13.26%)	Male	759 (57.50%)
38–47	230 (17.42%)	Asian	92 (6.97%)	Other/Non-binary	6 (0.45%)
48–57	135 (10.23%)	Hispanic/Latinx	89 (6.74%)	Missing	1 (0.08%)
58+	64 (4.85%)	Other	19 (1.44%)		
		American Indian/Alaska Native	12 (0.91%)		
		Hawaiian/Pacific Islander	1 (0.08%)		
		Missing	2 (0.15%)		

12. In Supplementary Table 11a, the upper confidence interval for Social Ideology was incorrectly rounded to 4.10, instead of 4.09.

The original incorrect confidence interval for Social Ideology reads ‘[−1.98, 4.10]’. The correct confidence interval for Social Ideology reads ‘[−1.98, 4.09]’

13. In Supplementary Table 11f, the confidence intervals for the Intercept, Gender, Income, and Economic Ideology were misreported.

The original table incorrectly stated confidence intervals for Intercept as ‘[72.94, 79.42]’, Gender as ‘[−2.53, .67]’, Income as ‘[−1.44, 1.98]’ and Economic Ideology as ‘[.61, 4.71]’.

The correct table states confidence intervals for Intercept as ‘[74.38, 79.52]’, Gender as ‘[−2.54, .65]’, Income as ‘[−1.1, 2.11]’ and Economic Ideology as ‘[.62, 4.73]’.

14. In Supplementary Table 15, the confidence intervals were misreported.

The original table incorrectly stated confidence intervals for Intercept as ‘[44.62, 50.89]’, Relational Norms as ‘[−5.23, .07]’, Social Closeness as ‘[2.98, 8.87]’, Interdependency as ‘[−2.32, 3.01]’, Genetic Relatedness as ‘[−1.95, 4.72]’, Action Likelihood as ‘[−6.70, .03]’ and Target Specificity as ‘[4.34, 9.75]’.

The correct table states confidence interval for Intercept as ‘[77.85, 128.4]’, Relational Norms as ‘[.10, .15]’, Social Closeness as ‘[−.42, .91]’, Interdependency as ‘[−.4, .44]’, Genetic Relatedness as ‘[−5.65, 7.9]’, Action Likelihood as ‘[−.69, −.34]’, and Target Specificity as ‘[.18, .86]’.

## Supplementary information

Corrected Supplementary information

Marked Up Supplementary information

Replication Script

