# Supplementary material for: How social relationships shape moral wrongness judgments
Source: Nat Commun. 2021 Oct 1;12:5776. doi: 10.1038/s41467-021-26067-4 (PMC8486868; doi:10.1038/s41467-021-26067-4)

Supplementary information for:

"How Social Relationships Shape Moral Wrongness Judgments"

Brian D. Earp, Killian L. McLoughlin, Joshua T. Monrad, Margaret S. Clark, and Molly J. Crockett

Table of contents

1. Stage 1 (p. 2)
1.1. Participants (p. 2)
    1.1.1. Supplementary Table 1: Summary of pre-registered exclusion criteria for Stage 1 (p. 2)
    1.1.2. Supplementary Table 2: Demographics of Sample 1 participants (p. 2)
1.2. Procedure (p. 3)
    1.2.1. Supplementary Table 3: Descriptions of relationship functions (p. 3)
    1.2.2. Supplementary Table 4: Descriptions of relationships (p. 5)
1.3. Data preparation and analysis details (p. 6)
1.4. Supplementary results (p. 7)
    1.4.1. Supplementary Table 5. Most to least functionally *polarized* relationships (p. 7)
    1.4.2. Supplementary Table 6. Most to least functionally *specific* relationships (p. 7)
    1.4.3. Supplementary Figure 1. Coalition ratings (p. 8)
    1.4.4. Supplementary Figure 2. Relational norm profiles: violin plots for 20 relationships (p. 9)
    1.4.5. Supplementary Tables 7a-7e. Complete demographic analyses: Stage 1/Sample 1 (p. 10)
2. Stage 2 (p. 13)
2.1. Materials creation (p. 13)
    2.1.1. Supplementary Table 8: Action statements used in Stage 2 (p. 14)
2.2. Participants (p. 15)
    2.2.1. Supplementary Table 9: Summary of exclusion criteria for Stage 2 (p. 15)
    2.2.2. Supplementary Table 10: Demographics of Sample 2 participants (p. 16)
2.3. Procedure (p. 16)
2.4. Data preparation and analysis details (p. 17)
2.5. Supplementary results (p. 17)
    2.5.1. Supplementary Tables 11a-11e. Complete demographic analyses: Stage 2/Sample 2 (p. 17)
    2.5.2. Supplementary Table 11f. Full regression table for the main analysis (p. 20)
    2.5.3. Supplementary Figures 3a-3d. Scatterplots of function-specific correlations (p. 21)
3. Stage 3 (p. 23)
3.1. Participants (p. 23)
    3.1.1. Supplementary Table 12: Summary of exclusion criteria for Stage 3 (p. 23)
    3.1.2. Supplementary Table 13: Demographics of Sample 3 participants (p. 23)
3.2. Procedure (p. 24)
    3.2.1. Supplementary Table 14: Dimensions of social closeness and interdependence (p. 24)
3.3. Data preparation and analysis details (p. 25)
3.4. Supplementary results (p. 25)
    3.4.1. Supplementary Table 15: Full-exclusion regression model for Stage 3 (p. 25)
    3.4.2. Supplementary Table 16: No exclusions regression model for Stage 3 (p. 25)
    3.4.3. Supplementary Figure 4: Study 3 results. Kernel density plot (p. 26)

1. Stage 1

Pre-registration #26400 on aspredicted.org. Full materials, raw data, and code available at https://osf.io/zxjt6/?view_only=66c1211300974dd68e97b88269fec4a3.

1.1. Participants

Using an online polling software (https://www.nbrii.com/our-process/sample-size-calculator/), we calculated that 385 participants would be required to have a representative U.S. sample with a 5% margin of error and 95% confidence level. Based on exclusion rates of previous studies conducted in our lab, we over-recruited by about 15% and recruited 450 U.S. participants representative for age, race, and gender via the Prolific Academic platform (Prolific). Ultimately 493 participants took some portion of the survey; not all of them finished. Participants were paid $0.60 to complete a training session in which they learned about the relational functions of care, coalition, hierarchy, reciprocity, and mating, plus a $2.00 bonus for completing the rest of the survey (rating each relationship on the extent to which it should ideally serve or not serve each function). To ensure high quality data, we included several attention, comprehension, and bot checks. Seventy (70) participants were excluded based on pre-registered exclusion criteria (see Supplementary Table 1). This left us with a final sample of 423 participants (217 female, 201 male, 4 other/nonbinary) ranging in age from 18 to 79 (M_{age} = 44.25, SD_{age} = 15.67); see Supplementary Table 2 for complete demographic information.

1.1.1. Supplementary Table 1: Summary of pre-registered exclusion criteria for Stage 1

Source data are provided as a Source Data file

**Exclusion criteria met**	**Type of check**	**Excluded *N***
Did not reach main portion of survey (i.e., did not pass training)	Comprehension check	10
Did not type in the word 'FRIDAY'	Bot check	6
Did not move slider to (at least) 1 of 2 specified positions	Attention check	54

Note: some participants met more than one criterion.

1.1.2. Supplementary Table 2: Demographics of Sample 1 participants

Source data are provided as a Source Data file

**Age**	**N (%)**	**Race**	**N (%)**	**Gender**	**N (%)**
18 - 27	82 (19.39%)	White	296 (69.98%)	Female	217 (51.30%)

28 - 37	78 (18.44%)	Black/ African-American	60 (14.18%)	Male	201 (47.52%)
38 - 47	70 (16.55%)	Asian	29 (6.86%)	Other/ Non-binary	4 (0.95%)
48 - 57	75 (17.73%)	Hispanic/Latinx	21 (4.96%)	Missing	1 (0.24%)
58+	117 (27.66%)	Other	10 (2.36%)		
Missing	1 (0.24%)	American Indian/ Alaska Native	4 (0.95%)		
		Hawaiian/ Pacific Islander	2 (0.47%)		
		Missing	1 (0.24%)		

1.2. Procedure

Participants completed a brief online survey through the Qualtrics interface. Before getting to the main part of the survey, participants were shown the full descriptions of each of the five main cooperative functions, as shown in Supplementary Table 3.

1.2.1. Supplementary Table 3: Descriptions of relationship functions

Function	Description
Care	**Full version:** The main purpose of this kind of relationship is to make sure that a person's basic well-being is secure, without any strings attached to the giving or receiving of support (like expecting compensation or favors in return, or feeling a debt). In other words, it is to make sure that people have someone in their corner on whom they can truly count for care and support, in good times and bad.<br><br>Note that there are two roles assumed by this relationship: the caregiving role (the person that can be truly counted on), and the care-seeking role (the person who may need unconditional support).<br><br>**Brief version:** the function of giving or receiving unconditional support
Hierarchy	**Full version:** The main purpose of this kind of relationship is to help coordinate behavior between people with different status (often they have unequal power or responsibility). In many situations, it is most effective for one person to be the 'leader' or have final say about what happens.<br><br>So, hierarchical relationships involve assigning people to different roles based on their status or power in a given situation, to help coordinate behavior and accomplish goals.

	There are two main roles in such relationships: the leader role and the follower role. The person in the leader role has 'final say' over what happens, while the person in the follower role ultimately must go along with what the leader decides.<br><br>**Brief version:** the function of coordinating behavior between people of different status, power, or responsibility
Mating	**Full version:** The main purpose of this kind of relationship is to find and maintain a sexual partner. For our ancestors, the ultimate point of mating was to produce healthy offspring, so that we could pass on our genes and continue as a species.<br><br>Of course, today we have birth control, and people often have sexual relationships without consciously planning to have children. But the underlying "logic" of the mating relationship -- in terms of the feelings and motivations it tends to inspire -- remains the same: to attract and secure a mate and stay with that person long enough to at least potentially have children together.<br><br>**Brief version:** the function of establishing and maintaining a sexual partnership
Reciprocity	**Full version:** The main purpose of this kind of relationship is to coordinate behavior between people who can mutually benefit, where they each have equal say in a given situation or activity.<br><br>So, this can be a kind of ‘tit-for-tat’ arrangement between people, where each one says, “I'll scratch your back if you scratch mine.” It can also be a way to keep things fair between people exchanging favors, goods, or services. It can even be a way to coordinate activities for mutual enjoyment, such as playing a game together. In any case, it involves making sure that the scales between people don't get too far out of balance.<br><br>**Brief version:** the function of coordinating behavior between people with equal say in a situation and keeping things fair
Coalition	**Full version:** The main purpose of this kind of relationship is to form and maintain a group identity, so group members can work toward a common goal. People in the same group look out for each other and try to promote their own group's interests over the interests of competing groups: “us versus them.”<br><br>This involves having shared expectations for what's normal or appropriate behavior and potentially making sacrifices for the good of the group, especially when it's in danger.<br><br>**Brief version:** the function of forming and maintaining a group identity for a common goal: us versus them

To ensure that participants were paying attention and were thinking of the functions in the way we wanted them to, each description was followed by a multiple-choice question about the definitions of the functions. Participants were not allowed to advance to the main part of the survey if they failed to answer this check correctly.

Once we had introduced the participants to the five cooperative functions, we gave them instructions for the main task of the survey. For each of 20 relationships, we asked participants how much the relationship ideally should serve each of the five cooperative functions. We specified that, by ‘ideally,’ we meant that “if this kind of relationship was the best possible relationship of its kind it could be,” how much should it serve each of those five functions?

Participants were then presented 20 blocks of questions, one for each relationship, in random order. For each relationship, we included a specific description of what we meant by that relationship – see Supplementary Table 4 for the descriptions. Then, for each combination of relationship and cooperative function, participants rated how much the relationship ideally should serve the given function, with the function now presented in its brief form as a reminder (see Supplementary Table 3). For instance, if the relationship and cooperative function pair was siblings/care, participants would be asked: "To what extent should the relationship between siblings ideally serve the function of **giving or receiving unconditional support**? (Care function.)" Responses were recorded on a sliding scale ranging from 'Definitely SHOULD NOT' (-100) through 'Neutral' (0) to 'Definitely SHOULD' (+100). Each of the recruited participants responded to questions about all five functions for all 20 relationships, yielding 100 data points per participant. Finally, we collected a battery of demographic measures: gender, age, race, ethnicity, income, level of education, English fluency, political leanings on social and economic issues, and religiosity.

1.2.2. Supplementary Table 4: Descriptions of relationships

Relationship	Description
Siblings	This refers to brothers and sisters. It includes adoptive as well as biological brothers and sisters.
Long-term romantic partners	This refers to romantic partners that have a commitment to each other, meaning they intend to remain together for the long term. It includes married partners like spouses, but also long-term romantic partners who aren't married but still have a commitment.
Close friends	This refers to people who seek one another out to spend time together, and who refer to each other as best friends, close friends, or good friends. It includes two people of any gender combination who are committed to remaining friends for the long term, and whose interest in each other is NOT primarily romantic.
Work colleagues or classmates	This refers to people who interact with each other on a regular basis at work/school or in work/school related activities. It includes only people who are on the same career or schooling level as each other.
Boss and employee	This refers to any workplace relationship in which one person directly supervises the other and that same person has some decision-making control over the other's activities and outcomes. It includes only people who interact with each other in person in a workplace.
Teacher and student	This refers to relationships in which someone with more experience guides someone with less experience, such as in school, sports or other extracurricular activity. It includes teacher-student or coach-player relationships, for example.
Doctor and patient	This refers to relationships in which one person provides expert medical or therapeutic services to the other person. It includes medical doctor-patient relationships or therapist-client relationships, for example.

Extended family members	This refers to relationships with relatives who are not in the immediate family (so, not parents or siblings). It includes relationships with a cousin, aunt, uncle, grandfather or grandmother.
Teammates	This refers to a relationship between two people who are in a clearly defined group together working towards a common goal. It includes teammates on a sports team or members of a theater troupe, for example.
Customer and seller	This refers to any relationship in which someone sells something and another person buys that thing, and they interact with each other directly. It includes a local baker and the baker's customer, or a house cleaner and the person who owns the house, for example.
Neighbors	This refers to people who live in the same section of an apartment building, or within the same block of houses on a street. It includes only people who know each other and at least occasionally interact.
Friends with benefits	This refers to people who know each other and interact sexually on a somewhat regular basis, but who refer to each other as friends rather than romantic partners. It includes friends who interact sexually without committing to a monogamous romantic relationship.
Acquaintances	This refers to people who know each other, and interact now and then, but don’t consider one another to be friends. It includes people one might know from work, school, or the neighborhood.
Members of a political party	This refers to people who are active, registered members of the same political party. They likely hold similar ideological positions and tend to vote for the same candidates in elections. They may not necessarily know each other for any other reason than their common political activities and party affiliation.
Roommates/housemates	This refers to anyone who lives together who are not family members or in a romantic relationship. This includes college room- or suite mates, apartment mates, or housemates.
Father and child (under 18)	This refers to fathers and their non-adult (meaning under age 18) children specifically. It includes both biological and adoptive fathers/children.
Mother and child (under 18)	This refers to mothers and their non-adult (meaning under age 18) children specifically. It includes both biological and adoptive parents/children.
Father and child (over 18)	This refers to fathers and their adult (meaning over age 18) children specifically. It includes both biological and adoptive fathers/children.
Mother and child (over 18)	This refers to mothers and their adult (meaning over age 18) children specifically. It includes both biological and adoptive mothers/children.
Strangers	This refers to people who encounter each other in any setting for the first time. It includes people who don’t know each other from another context and don't anticipate interacting again in the future.

1.3. Data preparation and analysis details

Raw data files (.csv) were prepared and analyzed using Python, within a Jupyter Notebook environment. Primary packages used: numpy, scipy, statsmodels, matplotlib, seaborn, pandas. For data files and all coding scripts, see the OSF link above.

1.4. Supplementary results

1.4.1. Supplementary Table 5. Most to least functionally *polarized* relationships

Source data are provided as a Source Data file

	Functional Expectations				
	Care	Hier.	Mate.	Recip.	Across all functions
	M	*M*	*M*	*M*	*SD*
Mother/child (under 18)	95.53	65.39	-98.22	9.05	85.33
Father/child (under 18)	93.99	67.39	-98.37	12.93	85.20
Siblings	84.06	-0.83	-98.47	74.96	84.64
Father/child (over 18)	86.58	35.97	-97.59	53.62	80.92
Mother/child (over 18)	88.05	28.02	-97.69	49.75	80.41
Extended family members	64.65	15.22	-96.40	57.26	74.32
Teacher and student	42.97	72.77	-95.61	24.49	73.90
Boss and employee	7.86	84.75	-92.17	29.14	73.87
Doctor and patient	53.75	41.63	-95.31	30.40	69.28
Close friends	79.39	-20.32	-50.31	79.96	67.51
Teammates	50.53	31.86	-73.00	75.43	65.29
Customer and seller	-18.37	22.04	-81.77	60.39	60.77
Roommates or housemates	24.90	-4.48	-52.39	87.30	58.42
Members of a political party	7.60	45.24	-66.04	63.82	57.44
Colleagues or classmates	17.38	18.92	-60.00	77.29	56.32
Neighbors	13.08	-16.25	-57.99	67.57	52.77
Romantic partners	92.43	-14.07	95.12	84.95	52.63
Friends with benefits	28.13	-30.36	58.43	59.87	42.21
Acquaintances	-2.74	-3.54	-46.82	51.38	40.21
Strangers	-26.62	-11.00	-55.87	34.95	37.93

1.4.2. Supplementary Table 6. Most to least functionally *specific* relationships

Source data are provided as a Source Data file

	Functional Expectations							
	Care	Hier.	Mate.	Recip.	Highest *M* Function	Highest *M* Value	Other Sum	Max Other Difference
	M	*M*	*M*	*M*		*M*		
Boss and employee	7.86	84.75	-92.17	29.14	Hierarchy	84.75	-55.17	139.92
Customer and seller	-18.37	22.04	-81.77	60.39	Reciprocity	60.39	-78.09	138.49
Neighbors	13.08	-16.25	-57.99	67.57	Reciprocity	67.57	-61.15	128.72

Strangers	-26.62	-11.00	-55.87	34.95	Reciprocity	34.95	-93.49	128.44
Mother/child (under 18)	95.53	65.39	-98.22	9.05	Care	95.53	-23.78	119.31
Roommates or housemates	24.90	-4.48	-52.39	87.30	Reciprocity	87.30	-31.96	119.25
Father/child (under 18)	93.99	67.39	-98.37	12.93	Care	93.99	-18.05	112.04
Siblings	84.06	-0.83	-98.47	74.96	Care	84.06	-24.34	108.40
Mother/child (over 18)	88.05	28.02	-97.69	49.75	Care	88.05	-19.91	107.96
Acquaintances	-2.74	-3.54	-46.82	51.38	Reciprocity	51.38	-53.09	104.47
Colleagues or classmates	17.38	18.92	-60.00	77.29	Reciprocity	77.29	-23.70	100.99
Teacher and student	42.97	72.77	-95.61	24.49	Hierarchy	72.77	-28.14	100.91
Father/child (over 18)	86.58	35.97	-97.59	53.62	Care	86.58	-8.00	94.59
Extended family members	64.65	15.22	-96.40	57.26	Care	64.65	-23.92	88.57
Doctor and patient	53.75	41.63	-95.31	30.40	Care	53.75	-23.29	77.04
Members of a political party	7.60	45.24	-66.04	63.82	Reciprocity	63.82	-13.20	77.02
Close friends	79.39	-20.32	-50.31	79.96	Reciprocity	79.96	8.77	71.19
Teammates	50.53	31.86	-73.00	75.43	Reciprocity	75.43	9.38	66.05
Friends with benefits	28.13	-30.36	58.43	59.87	Reciprocity	59.87	56.20	3.68
Romantic partners	92.43	-14.07	95.12	84.95	Mating	95.12	163.31	-68.18

1.4.3. Supplementary Figure 1. Coalition ratings. Kernel density plot of functional expectations for coalition only for 20 common relationships. Dot represents the mean; cap represents +/- 1 standard deviation. The height of the curve represents density: the likely proportions of scores (relative to each function) that fall within the given range along the x-axis. Source data are provided as a Source Data file.

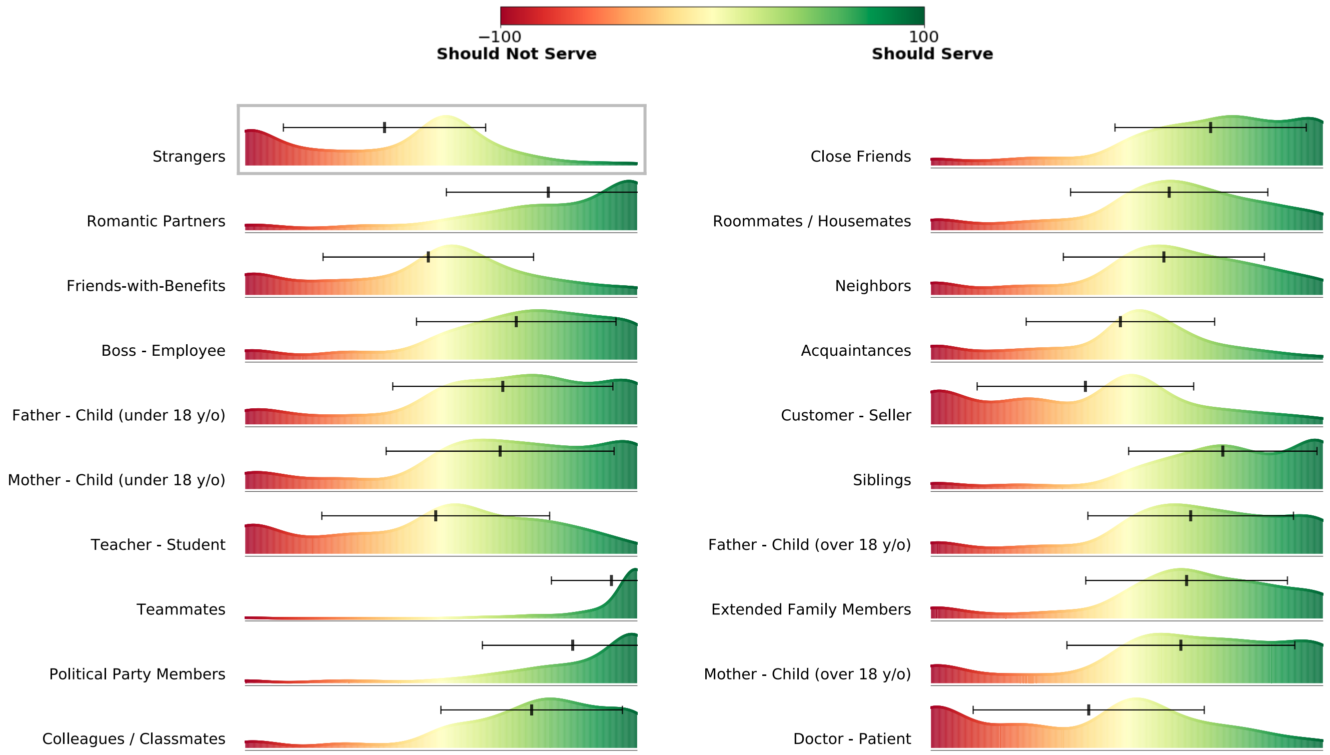

1.4.4. Supplementary Figure 2. Relational norm profiles: violin plots for all 20 relationships. Error bars represent the mean (dot) and +/- 1 SD (caps). Source data are provided as a Source Data file.

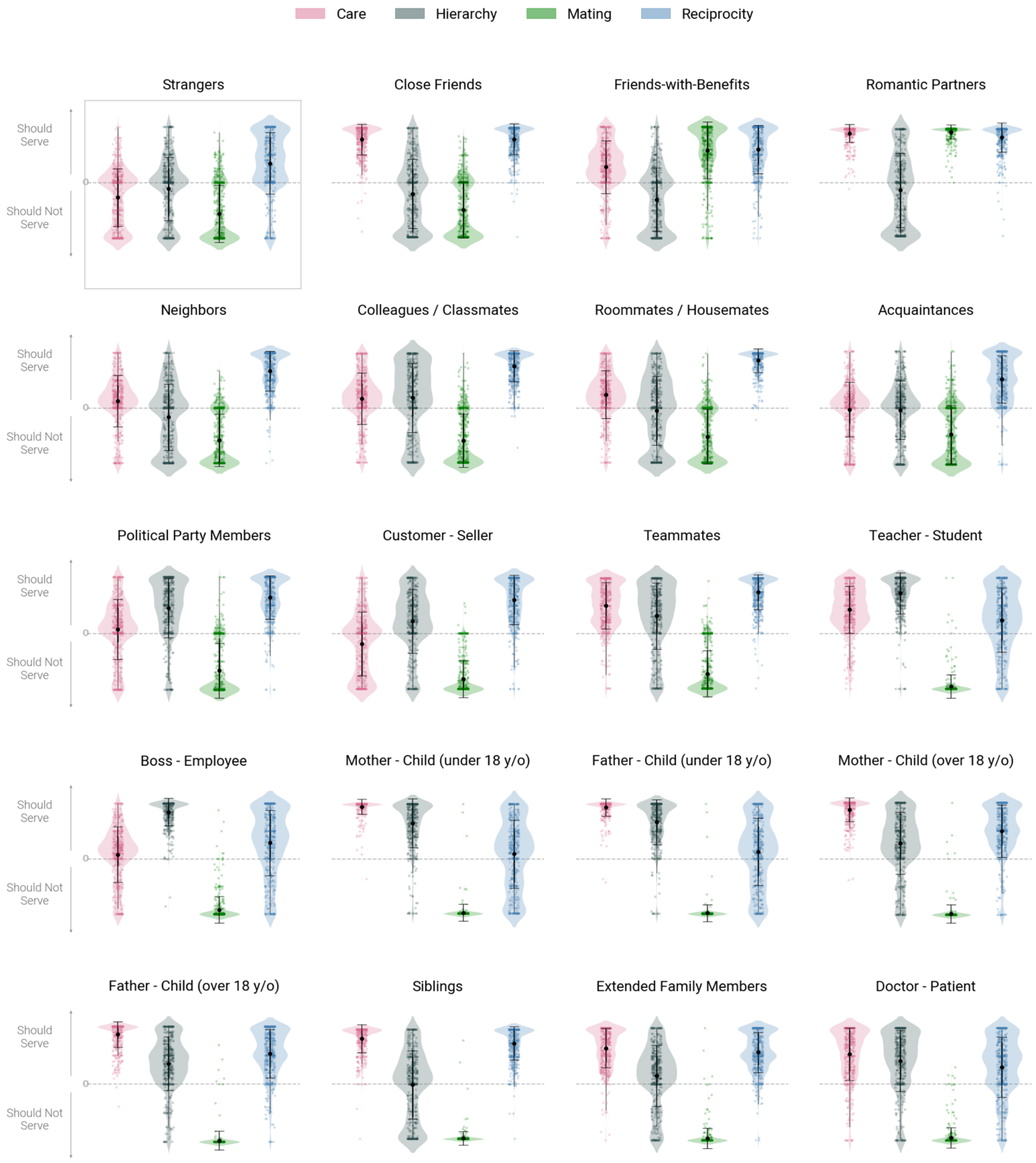

1.4.5. Supplementary Tables 7a-7e. Complete demographic analyses for Stage 1/Sample 1: regression tables. These regression tables show the results of the mixed effects linear regression models where participant and relationship are random factors, described in the main text. Note that participants who reported gender as 'other' were excluded from the analysis as this was not a big enough sub-group

to make statistical comparisons possible. Table a = overall model; b = gender and other demographic effects on functional expectations for mating; c = same for care; d = same for reciprocity; e = same for hierarchy. Source data are provided as a Source Data file.

a. Overall

Predictor	β	Std. Error	95% CI [LL, UL]	*p*	Fit
(Intercept)	13.74	1.00	[11.8, 15.69]	< .001	
Gender (Female)	1.00	.84	[-.66, 2.65]	.24	
Income (High)	-1.17	.82	[-2.79, .45]	.16	
Economic Ideology (Liberal)	-2.10	1.12	[-4.3, .10]	.06	
Social Ideology (Liberal)	2.49	1.14	[.27, 4.72]	.03	
Religiosity (Very Religious)	.61	.88	[-1.13, 2.34]	.49	
					Cond. R^2 = .0003

Note. *LL* and *UL* indicate the lower and upper limits of a 95% confidence interval, respectively. The same applies to all other relevant tables. Here, the coefficients and confidence intervals are based on the raw data, whereas the corresponding analyses reported in the main text used functional expectations scaled to each participant.

b. Mating

Predictor	β	Std. Error	95% CI [LL, UL]	*p*	Fit
(Intercept)	-57.42	1.66	[-60.67, -54.17]	< .001	
Gender (Female)	-7.15	1.40	[-9.9, -4.40]	< .001	
Income (High)	-3.61	1.37	[-6.30, -.92]	.01	
Economic Ideology (Liberal)	-5.65	1.87	[-9.32, -1.99]	.003	
Social Ideology (Liberal)	7.23	1.89	[3.52, 10.93]	< .001	

Religiosity (Very Religious)	-2.99	1.47	[-5.88, -.11]	.04	
					Conditional $R^2 = .8$

c. Care

Predictor	β	Std. Error	95% CI [LL, UL]	*p*	Fit
(Intercept)	39.84	1.5	[36.89, 42.78]	< .001	
Gender (Female)	6.36	1.27	[3.87, 8.86]	< .001	
Income (High)	-.57	1.24	[-3.01, 1.87]	.65	
Economic Ideology (Liberal)	-2.10	1.70	[-5.43, 1.22]	.22	
Social Ideology (Liberal)	.64	1.72	[-2.72, 4.00]	.71	
Religiosity (Very Religious)	5.05	1.33	[2.43, 7.66]	< .001	
					Conditional $R^2 = .8$

d. Reciprocity

Predictor	β	Std. Error	95% CI [LL, UL]	*p*	Fit
(Intercept)	51.10	1.33	[48.49, 53.71]	< .001	
Gender (Female)	4.80	1.13	[2.59, 7.01]	< .001	
Income (High)	-1.97	1.10	[-4.13, .19]	.07	
Economic Ideology (Liberal)	1.01	1.50	[-1.94, 3.95]	.50	
Social Ideology (Liberal)	.62	1.52	[-2.36, 3.60]	.68	
Religiosity	1.64	1.18	[-.68, 3.96]	.17	

(Very Religious)

Conditional $R^2 = .8$

e. Hierarchy

Predictor	β	Std. Error	95% CI [LL, UL]	*p*	Fit
(Intercept)	21.46	1.71	[18.10, 24.81]	< .001	
Gender (Female)	-.04	1.45	[-2.88, 2.80]	.98	
Income (High)	1.46	1.42	[-1.32, 4.24]	.30	
Economic Ideology (Liberal)	-1.65	1.93	[-5.44, 2.14]	.39	
Social Ideology (Liberal)	1.48	1.95	[-2.35, 5.31]	.45	
Religiosity (Very Religious)	-1.27	1.52	[-4.24, 1.71]	.41	
					Conditional $R^2 = .8$

2. Stage 2

Pre-registration #31592 on aspredicted.org. Full materials, raw data, and code available at https://osf.io/zxjt6/?view_only=66c1211300974dd68e97b88269fec4a3.

2.1. Materials creation

Selection of relationships

In Stage 1, we collected data for 20 different relationships. Our analysis of the Kolmogorov-Smirnov (K-S) distance between relationships (see the main text) revealed that several of these relationships were highly similar in terms of their prescribed cooperative functions (i.e., relational norm profiles). To avoid redundancy in Stage 2, we decided to use a subset of 10 relationships for the following study that were among the most functionally dissimilar to each other. Our procedure for selecting these relationships is described next.

As noted in the main manuscript, we first excluded the data pertaining to the coalition function. We then calculated the mean K-S distance score for every pairing of relationships across the four remaining functions. We set up 10 slots for "face-offs" between pairs of relationships with low K-S distances. For each face-off, we dropped the relationship with the lowest mean K-S distance from all other relationships (i.e., the more redundant of the two relationships considered in the context of the entire set). To enable gender comparisons, however, we first instituted a rule such that, if a father/mother relationship faced off, each was retained. Then, we moved sequentially from the lowest K-S score pairings to the highest K-S score pairings until all 10 slots were filled, dropping relationships along the way according to the first rule (if a relationship faced off with another relationship that had already been eliminated, it was retained by default). The final set of relationships identified by this method were: long-term romantic partners, friends with benefits, boss and employee, colleagues or classmates, mother/father and under-18 child, siblings, close friends, roommates or housemates, teammates, and strangers.

Selection of action statements

In this part of the study, we sought to test the hypothesis that the relational norm profile of a given relationship (based on the prescribed cooperative functions identified in Stage 1) would predict moral judgments for violations of associated functions in the context of that relationship. To this end, we created a set of 86 actions that we thought would plausibly weaken or impair one or more cooperative

functions. To determine the extent to which each action would characteristically weaken (or strengthen) each of the four dyadic cooperative functions, we had 15 trained judges rate each of the 86 actions in our set. These judges were recruited among lab members and colleagues and were given extensive training either in person or over Skype to ensure that their ratings reflected only the functional implications of each action (i.e., according to the cooperative logic of the functions) rather than being about moral judgments of any kind.

The trained judges completed an online survey, which included the same descriptions of cooperative functions that we used in Stage 1. After reading these descriptions and completing multiple comprehension checks, the judges were shown the 86 action statements, all of which were of the form "Person A does X to Person B" (see original materials at the OSF link above for the full list of actions). For each action, the judges were asked about the extent to which the action would weaken or strengthen each of the five functions. Their responses were recorded on a sliding scale, ranging from "Would characteristically **weaken**" (-100) through "It depends / Would neither weaken nor strengthen" (0) to "Would characteristically **strengthen**" (100). Thus, we obtained a mean rating between -100 and 100 for each action-function pair.

Based on the judges' ratings, we used an algorithm (described in the main text)[1] to identify 12 actions (three for each function) that were most characteristic as individual function-weakeners, while maintaining roughly equal "characteristicness" of items across functions.[2] This process resulted in a final set of 12 function-weakening action statements, depicted in Supplementary Table 8.

2.1.1. Supplementary Table 8: Action statements used in Stage 2

Function	Action
Care	- Person A sees Person B crying and walks away from them<br>- Person A keeps checking their cellphone while Person B tells a sad personal story<br>- Person A watches passively while Person B carries several heavy boxes up the stairs, even though they could easily help
Hierarchy	- Person A refuses to follow a reasonable order from Person B<br>- Person A repeatedly interrupts Person B while they are speaking<br>- Person A decides to skip a meeting scheduled with Person B without a good excuse
Mating	- Person A refuses to have sex with Person B<br>- Person A repeatedly turns down Person B's offer to go on a romantic date<br>- Person A invests time and energy in a romantic relationship with someone other than Person B

[1] MATLAB code is available at https://osf.io/j435q/.

[2] Three items from the 86-action set were excluded before running the algorithm due to having misleading or ambiguous wording. After running the code, we also dropped the item "Person A mocks Person B for a poor performance, when Person B tried their best," as we realized that the word "performance" could have different meanings across relationships (e.g., job performance versus an artistic performance).

Reciprocity	- Person A decides not to pay Person B back, hoping Person B won't remember<br>- Person A decides not to return Person B's nice favor<br>- Person A charges Person B $50 for an item worth $25.

2.2. Participants

In Stage 1, we powered for 95% confidence in a 5% margin of error for a nationally representative sample across age, race, and gender. This required that we have 385 observations per distribution (with each participant rating all 20 original relationships on all 5 original functions). To ensure that our Sample 2 distributions would be comparable to those from Sample 1, we powered for the same confidence in the same margin of error. In the first sample, every participant gave one rating per function for all of 20 relationships; in this study, Sample 2 participants would give three ratings for just one relationship out of a smaller set of 13 relationships (13 because questions regarding the non-symmetrical relationships, e.g., boss-employee, were asked in both directions). To achieve parity, then, we multiplied the previous target sample of 385 (per distribution) by 13 (accounting for the switch to a between-subjects design) and divided by three (accounting for three ratings per function in the current sample, compared to just one in Sample 1), yielding a required sample of 1,551. As in Stage 1, we over-recruited by about 10%, aiming for 1,706 participants for Stage 2. Ultimately, 1,822 participants took at least part of the survey (not all finished).

Once again, the participants were recruited online[3] and were paid were paid $1.00 to complete the survey. Five hundred and two (502) participants were excluded based on pre-registered exclusion criteria (see Supplementary Table 9 for criteria), leaving us with a final sample of 1,320 participants (554 female, 759 male, 6 other/non-binary, 1 missing) ranging in age from 18 to 73 (M_{age} = 35.33, SD_{age} = 10.58). See Supplementary Table 10 for complete demographic information.

2.2.1. Supplementary Table 9: Summary of exclusion criteria for Stage 2

Source data are provided as a Source Data file

Exclusion criteria met	Type of check	Excluded *N*
Failed question about survey instructions OR answered multiple-choice question incorrectly (correct answer includes the word "embarrassment")	Comprehension/attention check	246
Did not move slider to (at least) 1 of 2 specified position	Attention check	171

[3] Because Prolific did not allow for samples larger than 1,500, we instead relied on Amazon's Mechanical Turk platform (MTurk).

Failed CAPTCHA test	Bot check	0
Failed text-entry test	Bot check	53
Being younger than 18	Demographic check	2
Not fluent English speaker	Demographic check	12
Finished survey in < 4 min.	Quality check	73

Note: some participants met more than one criterion.

2.2.2. Supplementary Table 10: Demographics of Sample 2 participants

Source data are provided as a Source Data file

Age	N (%)	Race	N (%)	Gender	N (%)
18 - 27	329 (24.92%)	White	930 (70.45%)	Female	554 (41.97%)
28-37	562 (42.58%)	Black/ African-American	175 (13.26%)	Male	759 (57.50%)
38-47	230 (17.42%)	Asian	92 (6.97%)	Other/ Non-binary	6 (0.45%)
48-57	135 (10.23%)	Hispanic/ Latinx	89 (6.74%)	Missing	1 (0.08%)
58+	64 (4.85%)	Other	19 (1.44%)		
		American Indian/ Alaska Native	12 (0.91%)		
		Hawaiian/ Pacific Islander	1 (0.08%)		
		Missing	2 (0.15%)		

2.3. Procedure

Each participant was assigned to one of 13 relationship-pairs (13 because questions about the non-symmetrical relationships, e.g., boss-employee, were asked separately in both directions), and were shown a brief description of their assigned relationship (see Supplementary Table 4). We informed

participants that they would be asked to consider various actions in the context of their assigned relationship and to answer how morally wrong each of those actions would be. To orient them to the rating scale, we clarified that none of the actions they would see would be extreme (e.g., murder), but rather would all be actions that might plausibly occur within the course of day-to-day life. We then 'anchored' their expectations by showing them a list of actions comparable to the ones included in the task.

Following instructions and attention checks, participants were shown the 12 (three for each of the four functions) hypothetical actions selected by our algorithmic approach described above. For instance, for the sibling relationship, participants were asked, e.g., "Imagine that someone keeps checking their cellphone while their sibling tells a sad personal story. How morally wrong would that be, if at all?" Responses were recorded on a sliding scale from "Not at all morally wrong" (0) to "Very morally wrong" (100). Finally, we collected exploratory data about how likely it is that each action would occur in real life, plus the same demographic measures that we collected from Sample 1 participants.

2.4. Data preparation and analysis details

Raw data files (.csv) were prepared and analyzed using Python, within a Jupyter Notebook environment. Primary packages used: numpy, scipy, statsmodels, matplotlib, seaborn, pandas. For data files and all coding scripts, see the OSF link above.

2.5. Supplementary results

2.5.1. Supplementary Tables 11a-11e. Complete demographic analyses for Stage 2/Sample 2: regression tables. These regression tables show the results of the mixed effects linear regression models where participant and relationship are random factors, described in the main text. Note that participants who reported gender as 'other' were excluded from the analysis as this was not a big enough sub-group to make statistical comparisons possible. For each set of regression results below, an Anderson-Darling test indicated that the outcome variable was non-normally distributed (all *p*s < .001). However, Q-Q plots of standardized residuals against fitted values indicated that this did not impact model performance.[4] Table a = overall model; b = gender and other demographic effects on moral wrongness judgments for mating; c = same for care; d = same for reciprocity; e = same for hierarchy. Source data are provided as a Source Data file.

[4] The Q-Q plot (quantile-quantile plot) is a visualization that assesses whether data plausibly came from a theoretical distribution, such as a normal distribution. Q-Qs can be used to inspect whether the assumption of a normally distributed outcome measure required by certain statistical tests is violated. The graph plots two sets of quantiles or percentiles – thresholds below which certain points of our data fall – against one another: the observed quantiles and those of the theoretical (normal) distribution. If both sets of quantiles came from the same distribution, the points should form a line that is roughly straight. In the case of our linear mixed effects model predicting moral wrongness judgments from functional expectations, the Q-Q plot forms a nearly perfect straight line (see the code listed at https://osf.io/zxjt6/). This suggests that the observed non-normality of our dependent measure did not violate the normality assumption of the model. Gelman and Hill (2007) note that the normality or otherwise of residuals doesn't affect the parameter estimates in multilevel models. They therefore advise against normality tests of regression residuals (p. 46). Gelman, A., Hill, J., 2007. *Data Analysis Using Regression and Multilevel/Hierarchical Models*. Cambridge University Press.

a. Overall

Predictor	β	Std. Error	95% CI [LL, UL]	*p*	Fit
(Intercept)	39.85	1.35	[37.21, 42.50]	< .001	
Gender (Female)	-.12	1.22	[-2.51, 2.28]	.92	
Income (High)	1.49	1.20	[-.87, 3.85]	.22	
Economic Ideology (Liberal)	-.48	1.54	[-3.50, 2.53]	.75	
Social Ideology (Liberal)	1.06	1.55	[-1.98, 4.09]	.50	
Religiosity (Very Religious)	8.93	1.22	[6.54, 11.32]	< .001	
					Conditional $R^2 = .07$

b. Mating

Predictor	β	Std. Error	95% CI [LL, UL]	*p*	Fit
(Intercept)	-.07	1.83	[-3.66, 3.52]	.97	
Gender (Female)	-2.94	1.66	[-6.19, .31]	.08	
Income (High)	5.09	1.63	[1.89, 8.29]	.002	
Economic Ideology (Liberal)	2.00	2.09	[-2.09, 6.10]	.34	
Social Ideology (Liberal)	3.17	2.10	[-.95, 7.30]	.13	
Religiosity (Very Religious)	16.53	1.66	[13.28, 19.78]	< .001	
					Conditional $R^2 = .59$

c. Care

Predictor	β	Std. Error	95% CI [LL, UL]	*p*	Fit

(Intercept)	57.20	2.73	[51.85, 62.55]	< .001	
Gender (Female)	3.51	2.49	[-1.37, 8.39]	.16	
Income (High)	.24	2.43	[-4.53, 5.01]	.92	
Economic Ideology (Liberal)	1.65	3.49	[-5.20, 8.50]	.64	
Social Ideology (Liberal)	-.19	3.52	[-7.09, 6.71]	.96	
Religiosity (Very Religious)	3.52	2.45	[-1.29, 8.32]	.15	
					Conditional R^2 = .41

d. Reciprocity

Predictor	β	Std. Error	95% CI [LL, UL]	*p*	Fit
(Intercept)	64.27	1.63	[61.08, 67.46]	< .001	
Gender (Female)	.26	1.47	[-2.63, 3.15]	.86	
Income (High)	-.95	1.45	[-3.79, 1.89]	.51	
Economic Ideology (Liberal)	-2.04	1.86	[-5.68, 1.60]	.27	
Social Ideology (Liberal)	.20	1.87	[-3.46, 3.86]	.91	
Religiosity (Very Religious)	4.01	1.47	[1.13, 6.90]	.006	
					Conditional R^2 = .07

e. Hierarchy

Predictor	β	Std. Error	95% CI [LL, UL]	*p*	Fit
(Intercept)	53.76	3.07	[47.74, 59.78]	< .001	

Gender (Female)	1.51	2.77	[-3.91, 6.93]	.59	
Income (High)	.75	2.74	[-4.61, 6.11]	.78	
Economic Ideology (Liberal)	-3.78	3.23	[-10.12, 2.55]	.24	
Social Ideology (Liberal)	-1.34	3.24	[-7.69, 5.01]	.68	
Religiosity (Very Religious)	8.44	2.81	[2.95, 13.94]	.003	
					Conditional $R^2 = .61$

2.5.2. Supplementary Table 11f. Full regression table for the main analysis, controlling for demographic information (i.e., the results of the mixed effects linear regression model described in the main text). Source data are provided as a Source Data file.

Predictor	β	Std. Error	95% CI [LL, UL]	*p*	Fit
(Intercept)	76.95	1.31	[74.38, 79.52]	< .001	
Relational Norms	16.26	.32	[15.64, 16.88]	< .001	
Action Likelihood	-.20	.01	[-.21, -.18]	< .001	
Target Specificity	.37	.01	[.34, .40]	<.001	
Gender (Female)	-.94	.81	[-2.54, .65]	.25	
Income (High)	.50	.82	[-1.1, 2.11]	.54	
Economic Ideology (Liberal)	2.67	1.05	[.62, 4.73]	.01	
Social Ideology (Liberal)	-1.86	1.05	[-3.92, .21]	.08	
Religiosity (Very Religious)	9.16	.82	[7.55, 10.77]	< .001	
					Conditional $R^2 = .63$

2.5.3. Supplementary Figures 3a-3d. Scatterplots of function-specific correlations between KS distance scores in relational norm and moral judgment space respectively: a = care, b = mating, c = hierarchy, d = reciprocity. Spearman's r is the reported value. For the legend describing the relationship labels and colors, please see the main text. Source data are provided as a Source Data file.

a.

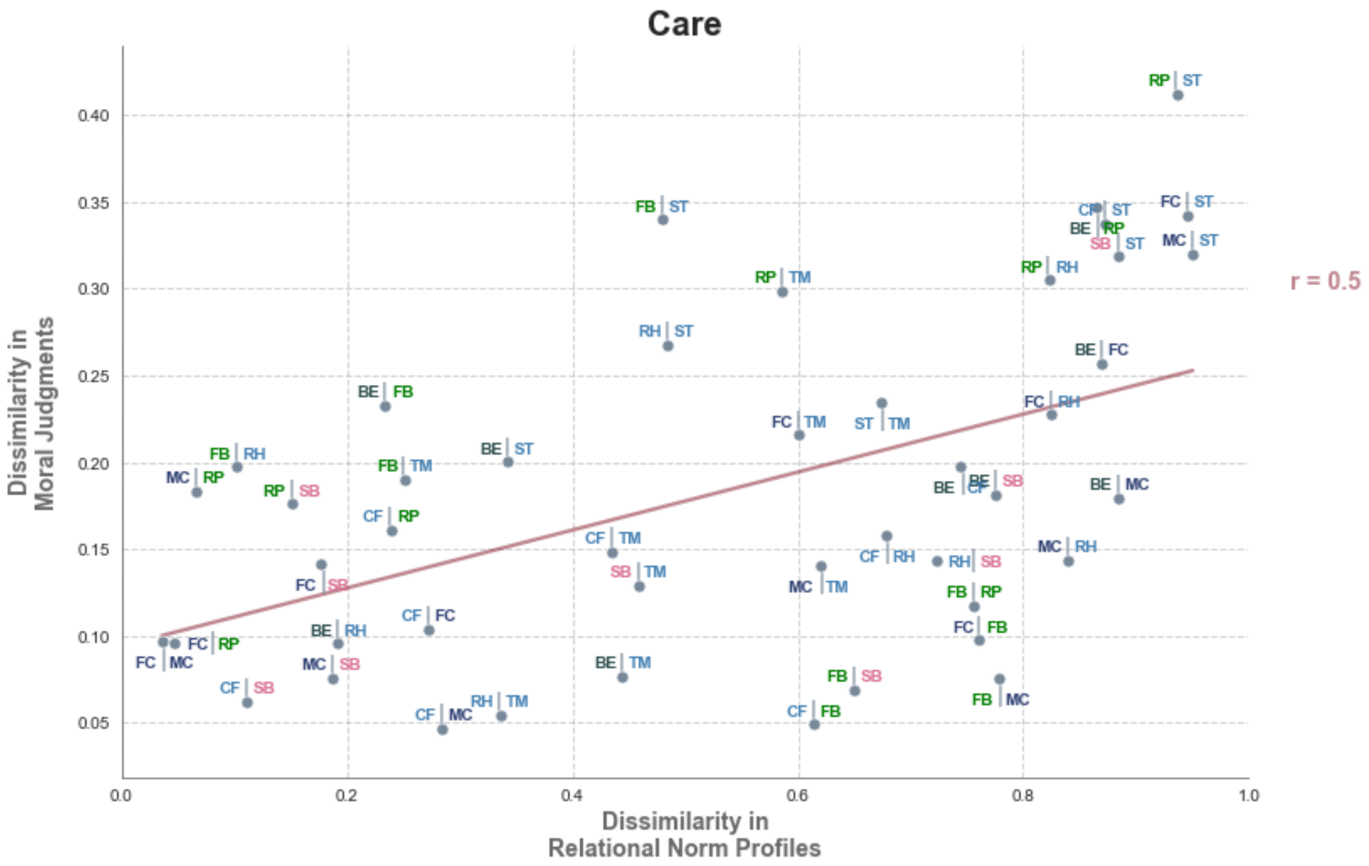

b.

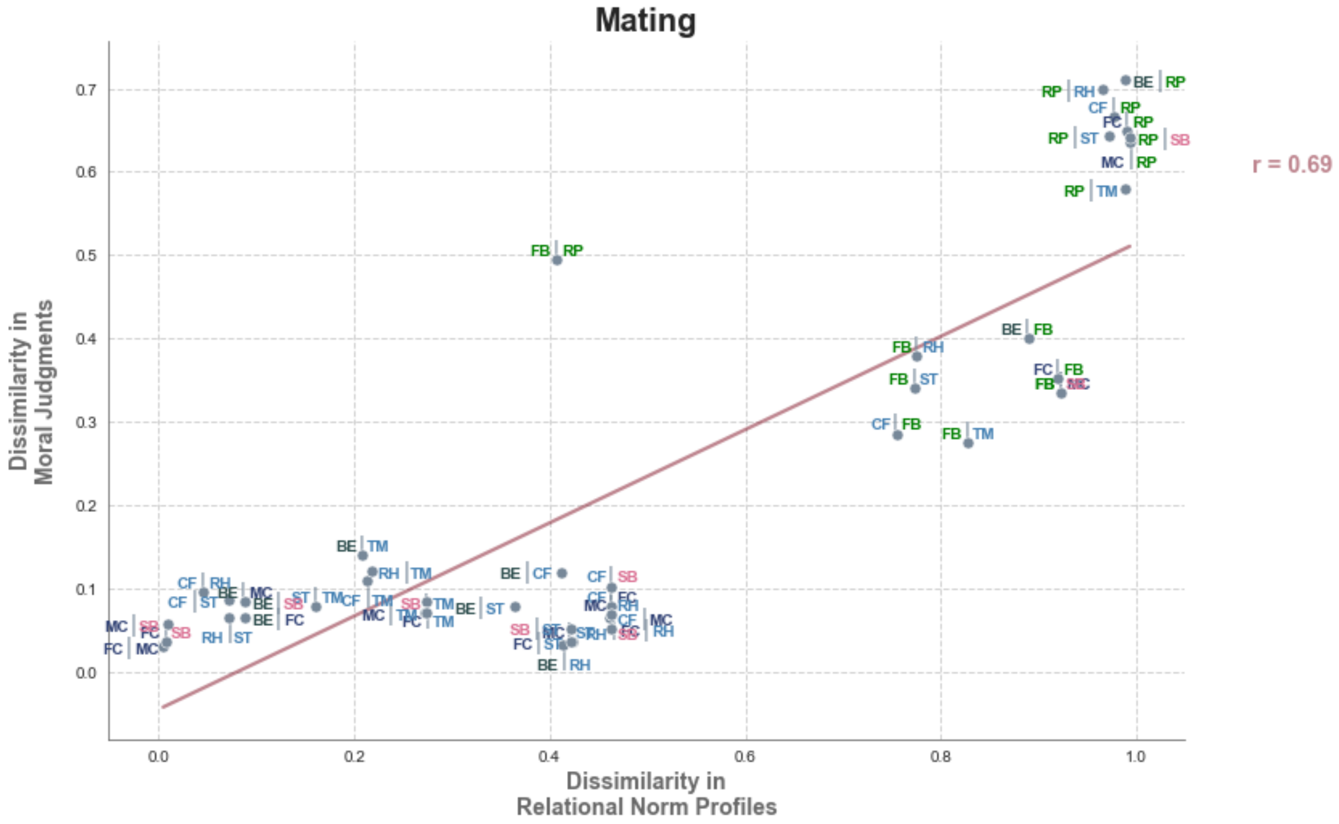

c.

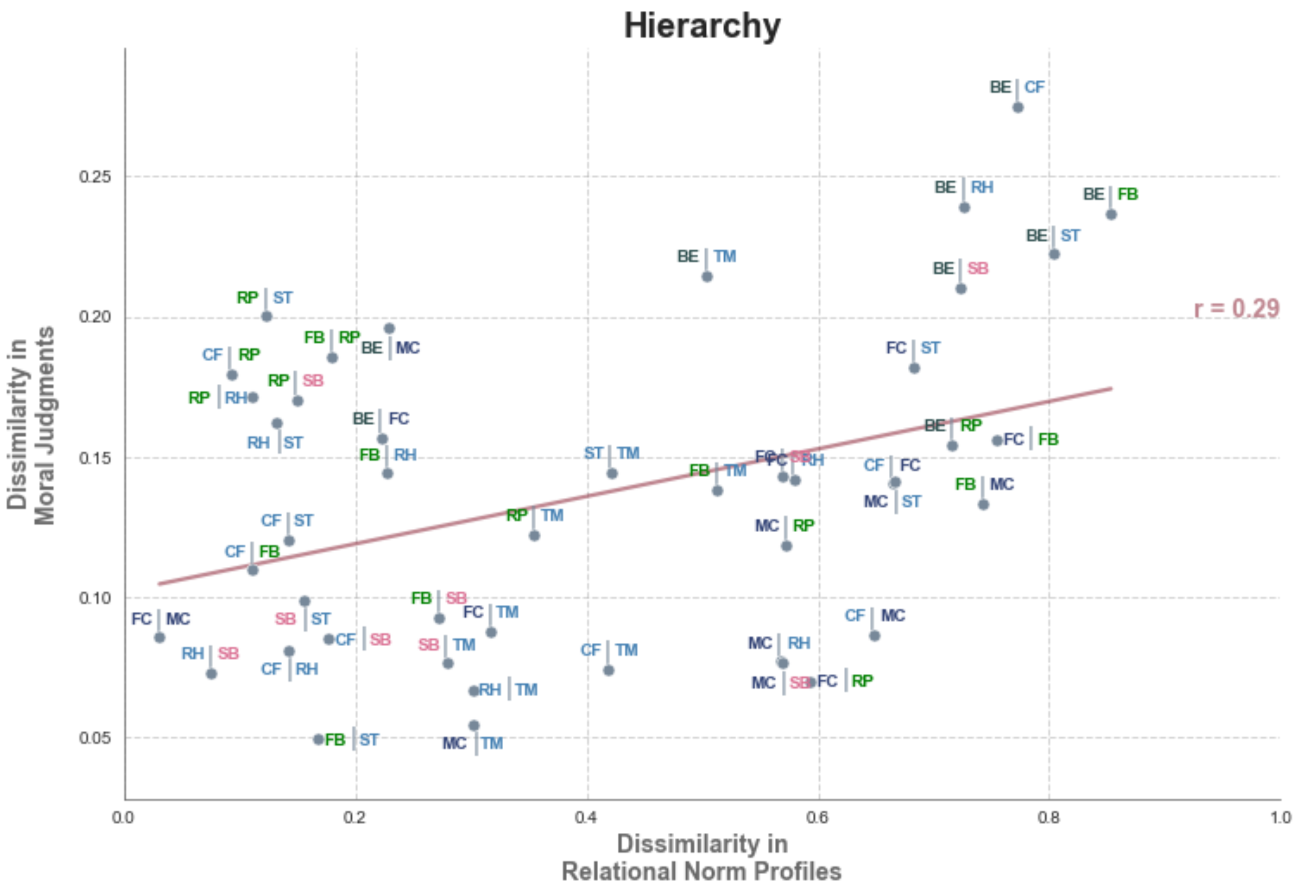
Hierarchy
r = 0.29
Dissimilarity in
Moral Judgments
Dissimilarity in
Relational Norm Profiles

d.

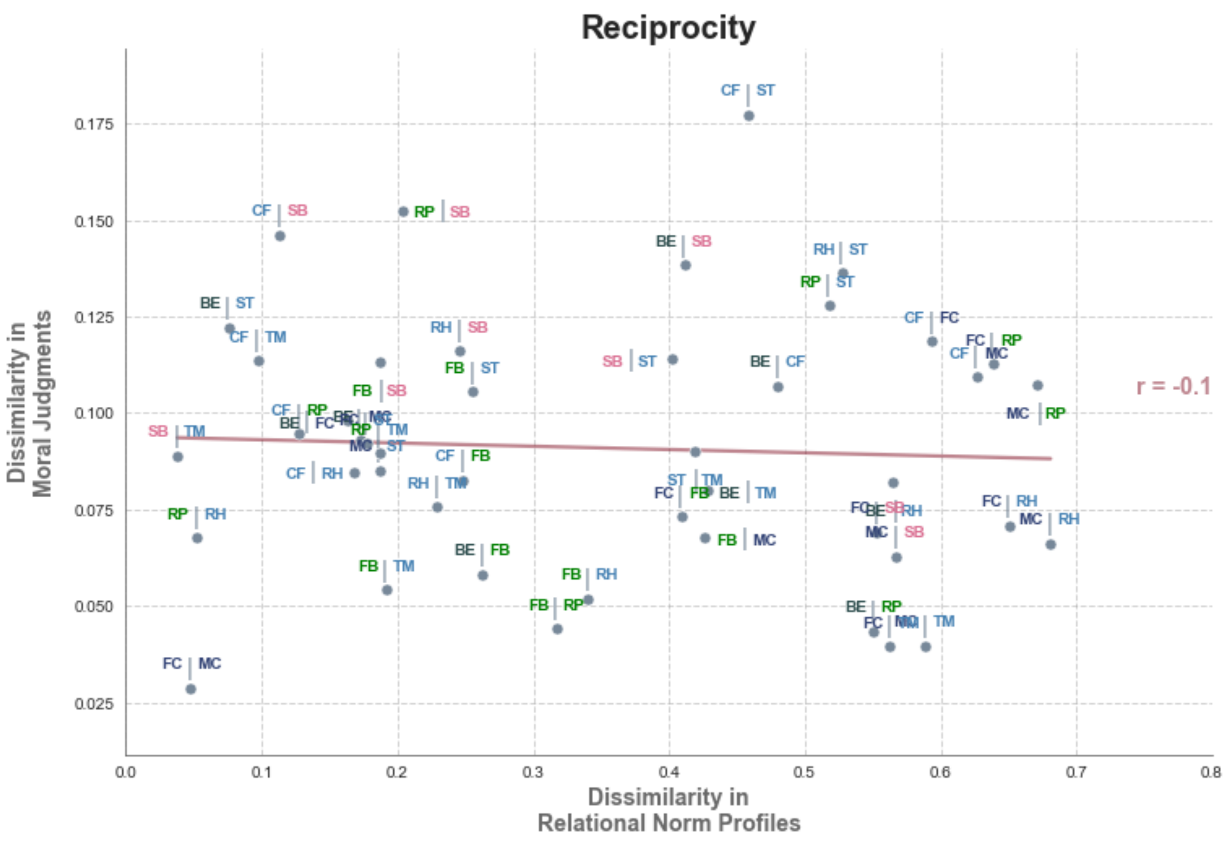
Reciprocity
r = -0.1
Dissimilarity in
Moral Judgments
Dissimilarity in
Relational Norm Profiles

3. Stage 3

3.1. Participants

For Stage 3, we aimed to have as many observations per distribution as we had in Stage 1. For Stage 1, which was powered for a nationally representative sample across age, race, and gender, we recruited for 450 observations with a final sample of 423 (Sample 1). In the current study, each participant would have to make three ratings for each measured construct rather than one (as in Stage 1), so we determined to recruit a third of the number of target participants as we had in our first sample to achieve similar statistical power. Accordingly, we recruited 150 participants, with 167 participants ultimately taking at least part of the survey (not all finished). Each participant who completed the survey[5] was paid $1.00. Sixty-four participants (64) were excluded on the basis of the pre-registered exclusion criteria (see Supplementary Table 12), leaving us with a final sample of N = 85 (38 female, 46 male, 1 other/non-binary) ranging in age from 21 to 69 (M_{age} = 34.82, SD_{age} = 10.66); see Supplementary Table 13 for complete demographic information.

3.1.1. Supplementary Table 12: Summary of exclusion criteria for Stage 3

Source data are provided as a Source Data file

**Exclusion criteria met**	**Type of check**	**Excluded *N***
Did not move sliders to (at least) 1 of 2 specified positions	Attention check	64

3.1.2. Supplementary Table 13: Demographics of Sample 3 participants

Source data are provided as a Source Data file

**Age**	**N (%)**	**Race**	**N (%)**	**Gender**	**N (%)**
18 - 27	23 (27.06%)	White	59 (69.41%)	Female	38 (44.71%)
28 - 37	37 (43.53%)	Black/ African-American	12 (14.12%)	Male	46 (54.12%)
38 - 47	14 (16.47%)	Asian	5 (5.88%)	Other/ Non-binary	1 (1.18%)
48 - 57	6 (7.06%)	Hispanic/ Latinx	4 (4.71%)		

[5] 18 “participants” in the raw data file had no location information recorded and the data posted to Qualtrics nearly a week after the completion of the survey. We assumed these were bots and deleted those 18 lines of data prior to applying the exclusion criterion. Hence the n = 149 “completed” participants reported in the manuscript: 167-18 = 149.

58+	5 (5.88%)	Multiracial	3 (3.53%)
		Native American	2 (2.35%)

3.2. Procedure

We informed participants that they would be shown a set of relationships and asked some questions about each. Then, participants were shown, in random order, each of the 10 relationship pairs that were used in Stage 2 along with a brief description. Because this study was conducted during the COVID-19 pandemic, we were concerned that participants' judgments about relationships might be affected by the unprecedented circumstances. To address this concern, we included a note asking participants to think of the relationships as they would be under more 'normal' circumstances.

For each of the 10 relationship pairs, participants were asked to rate it along three dimensions of social closeness and three dimensions of interdependence: see Supplementary Table 14 for the exact descriptions of each. As an example, for the sibling relationship and one of the social closeness dimensions, participants were asked, e.g., "In an ideal, well-functioning relationship between siblings, to what extent would the relationship be characterized by **deeply understanding each other**?" Responses were recorded on a sliding scale from 0 to 100 (see endpoint labels in Supplementary Table 14 below). Finally, we included similar individual demographic measures as in the previous samples.

3.2.1. Supplementary Table 14: Dimensions of social closeness and interdependence

Construct	Dimension
Social Closeness	- In an ideal, well-functioning relationship between [relationship pair], to what extent would the relationship be characterized by **deeply understanding each other**? (0 = Not at all; 100 = A great deal)<br><br>- In an ideal, well-functioning relationship between [relationship pair], to what extent would the relationship be characterized by **accepting and validating each other's natures**? (0 = Not at all; 100 = A great deal)<br><br>- In an ideal, well-functioning relationship between [relationship pair], to what extent would the relationship be characterized by **striving to care for and promote each other's overall well-being**? (0 = Not at all; 100 = A great deal)
Inter-dependence	- In an ideal, well-functioning relationship between [relationship pair], how **frequently** would they affect each other's thoughts, feelings, and behaviors? (0 = Not at all frequently; 100 = Very frequently)<br><br>- In an ideal, well-functioning relationship between [relationship pair], in **how many different ways** would they affect each other's thoughts, feelings, and behaviors across different situations? (0 = Very few ways; 100 = A great variety of ways)<br><br>- In an ideal, well-functioning relationship between [relationship pair], how **strongly** would they affect each other's thoughts, feelings, and behaviors? (0 = Not at all strongly; 100 = Very strongly)

3.3. Data preparation and analysis details

Raw data files (.csv) were prepared and analyzed using Python, within a Jupyter Notebook environment. Primary packages used: numpy, scipy, statsmodels, matplotlib, seaborn, pandas. For data files and all coding scripts, see the OSF link above.

3.4. Supplementary results

3.4.1. Supplementary Table 15: Full-exclusion regression model for Stage 3. This table shows the full results for the mixed effects linear regression model described in the main text. Note: because there are three samples, the data are not at the participant level, which precludes controlling for demographic information. Source data are provided as a Source Data file.

Predictor	β	Std. Error	95% CI [LL, UL]	*p*	Fit
(Intercept)	103.12	13.15	[77.85, 128.4]	< .001	
Relational Norms	.12	.01	[.10, .15]	< .001	
Social Closeness	.24	.35	[-.42, .91]	.48	
Interdependency	-.20	.33	[-.4, .44]	.55	
Genetic Relatedness	1.13	3.52	[-5.65, 7.9]	.75	
Action Likelihood	-.51	.09	[-.69, -.34]	< .001	
Target Specificity	.52	.18	[.18, .86]	< .01	Conditional R^2 = .89

3.4.2. Supplementary Table 16: No exclusions regression model for Stage 3. Full results for the mixed effects linear regression model described in the main text, but with no exclusions. Source data are provided as a Source Data file.

Predictor	β	Std. Error	95% CI [LL, UL]	*p*	Fit
(Intercept)	104.38	15.54	[74.49, 134.26]	< .001	
Relational Norms	9.36	.96	[7.51, 11.21]	< .001	
Social Closeness	.44	.73	[-.96, 1.84]	.55	
Interdependency	-.36	.70	[-1.71, .1]	.61	
Genetic Relatedness	.89	3.41	[-5.67, 7.46]	.79	
Action Likelihood	-.53	.09	[-.70, -.36]	< .001	
Target Specificity	.54	.18	[.19, .88]	< .01	Conditional R^2 = .90

3.4.3. Supplementary Figure 4: Study 3 results. Kernel density plot of expectations for social closeness and interdependency for 10 common relationships. Dot represents the mean, with caps representing +/- 1 standard deviation. The height of the curve represents density: the likely proportions of scores (relative to each function) that fall within the given range along the x-axis. Source data are provided as a Source Data file.

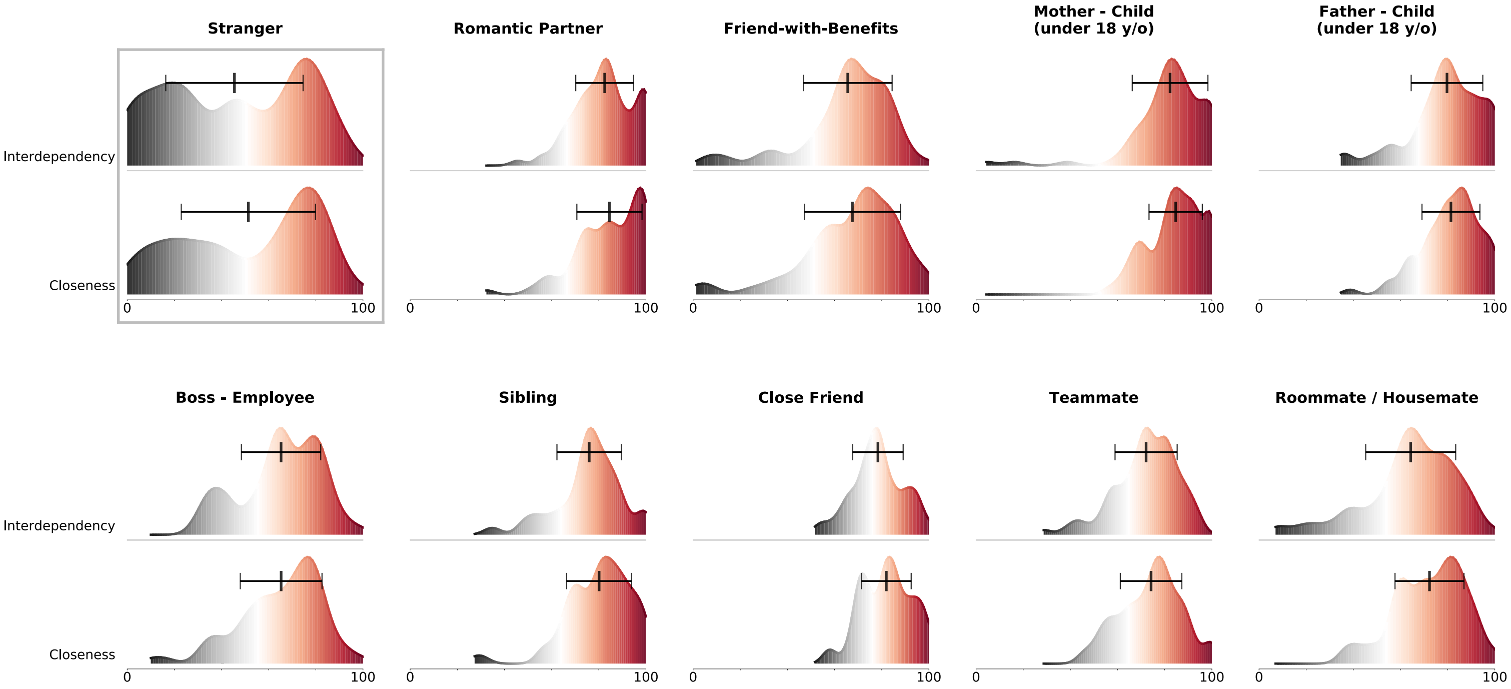

Supplement: Supplementary file 1 — Supplementary Information [file 41467_2021_26067_MOESM1_ESM.pdf]
