# Supplementary material for: How social relationships shape moral wrongness judgments
Source: Nat Commun. 2021 Oct 1;12:5776. doi: 10.1038/s41467-021-26067-4 (PMC8486868; doi:10.1038/s41467-021-26067-4)
Supplement: Supplementary file 3 — Reporting Summary [file 41467_2021_26067_MOESM3_ESM.pdf]

nature research

Corresponding author(s): Brian D. Earp and Molly J. Crockett

Last updated by author(s): 2021/5/1

Reporting Summary

Nature Research wishes to improve the reproducibility of the work that we publish. This form provides structure for consistency and transparency in reporting. For further information on Nature Research policies, see our Editorial Policies and the Editorial Policy Checklist.

Statistics

For all statistical analyses, confirm that the following items are present in the figure legend, table legend, main text, or Methods section.

n/a	Confirmed	
☐	☒	The exact sample size (*n*) for each experimental group/condition, given as a discrete number and unit of measurement
☐	☒	A statement on whether measurements were taken from distinct samples or whether the same sample was measured repeatedly
☐	☒	The statistical test(s) used AND whether they are one- or two-sided<br>*Only common tests should be described solely by name; describe more complex techniques in the Methods section.*
☐	☒	A description of all covariates tested
☐	☒	A description of any assumptions or corrections, such as tests of normality and adjustment for multiple comparisons
☐	☒	A full description of the statistical parameters including central tendency (e.g. means) or other basic estimates (e.g. regression coefficient) AND variation (e.g. standard deviation) or associated estimates of uncertainty (e.g. confidence intervals)
☐	☒	For null hypothesis testing, the test statistic (e.g. F, t, r) with confidence intervals, effect sizes, degrees of freedom and P value noted<br>*Give P values as exact values whenever suitable.*
☒	☐	For Bayesian analysis, information on the choice of priors and Markov chain Monte Carlo settings
☐	☒	For hierarchical and complex designs, identification of the appropriate level for tests and full reporting of outcomes
☐	☒	Estimates of effect sizes (e.g. Cohen's *d*, Pearson's *r*), indicating how they were calculated

Our web collection on statistics for biologists contains articles on many of the points above.

Software and code

Policy information about availability of computer code

Data collection: All data collected via either the MTurk or Prolific platforms, using a survey designed in the Qualtrics platform

Data analysis: Raw data files (.csv) were prepared and analyzed using Python (version 3.7.4), within a Jupyter Notebook (version 7.8.0) environment. Primary packages used: numpy (version 1.17.2), scipy (version 1.3.1), statsmodels (version 0.10.1), matplotlib (version 3.1.2), sea born (version 0.11.0), pandas (version 0.25.1). The linear mixed effect models are modeled in R (version 4.0.3 [2020-10-10]) using packages nlme (version 3.1.149), MuMIn (version 1.43.17), and lattice (version 0.20.41). The online polling software used to determine sample size for the nationally representative samples was https://www.nbrii.com/our-process/sample-size-calculator/ (version 2021). The analysis in MATLAB was done in version 9.7 R2019b). For data files and all coding scripts, see the OSF link associated with the paper: https://osf.io/zxjt6/.

For manuscripts utilizing custom algorithms or software that are central to the research but not yet described in published literature, software must be made available to editors and reviewers. We strongly encourage code deposition in a community repository (e.g. GitHub). See the Nature Research guidelines for submitting code & software for further information.

Data

Policy information about availability of data

All manuscripts must include a data availability statement. This statement should provide the following information, where applicable:

- Accession codes, unique identifiers, or web links for publicly available datasets
- A list of figures that have associated raw data
- A description of any restrictions on data availability

All original data (anonymized) is available open access at https://osf.io/zxjt6/. Source data for figures can be found in the folder labeled "Source Data - Figures."

Field-specific reporting

Please select the one below that is the best fit for your research. If you are not sure, read the appropriate sections before making your selection.

☐ Life sciences ☒ Behavioural & social sciences ☐ Ecological, evolutionary & environmental sciences

For a reference copy of the document with all sections, see nature.com/documents/nr-reporting-summary-flat.pdf

Behavioural & social sciences study design

All studies must disclose on these points even when the disclosure is negative.

Study description

Quantitative data from a U.S. nationally representative survey used to predict moral judgments in a separate convenience sample using a linear mixed regression model.

Research sample

Sample 1: US sample nationally representative for age, race, and gender; data collected via Prolific online platform: specific breakdowns are in table S2 of the manuscript and reproduced below. Samples 2 and 3: US convenience samples, data collected via the MTurk platform; specific breakdowns for age, race, gender, and other demographics are in tables S10 and S13 respectively, and reproduced below. The nationally representative Sample 1 was chosen because we wanted to establish population-level norms for functional expectations within U.S. society. The convenience samples were chosen because (for Sample 2) MTurk allows a larger sample size collection and we needed to collect more data than is allowed on Prolific due to the between-subjects study design employed; and for Sample 3, a convenience sample was chosen due to funding constraints.

Sample 1 (nationally representative for age, race, gender), demographic breakdown:

Age N (%)
18 - 27 82 (19.39%)
28 - 37 78 (18.44%)
38 - 47 70 (16.55%)
48 - 57 75 (17.73%)
58+ 117 (27.66%)

Race N (%)
White 296 (69.98%)
Black/African-American 60 (14.18%)
Asian 29 (6.86%)
Hispanic/Latinx 21 (4.96%)
Other 10 (2.36%)
Missing 1 (0.24%)
American Indian/Alaska Native 4 (0.95%)
Hawaiian/Pacific Islander 2 (0.47%)

Gender N (%)
Female 217 (51.30%)
Male 201 (47.52%)
Other/Non-binary 4 (0.95%)

Sample 2 (MTurk convenience sample, NOT nationally representative), demographic breakdown:

Age N (%)
18 - 27 329 (24.96%)
28-37 561 (42.56%)
38-47 230 (17.45%)
48-57 135 (10.24%)
58+ 63 (4.78%)

Race N (%)
White 929 (70.49%)
Black/African-American 175 (13.28%)
Asian 92 (6.98%)
Hispanic/Latinx 88 (6.68%)
Other 19 (1.44%)
American Indian/Alaska Native 12 (0.91%)
Hawaiian/Pacific Islander 1 (0.08%)

Gender N (%)
Female 553 (41.96%)
Male 758 (57.51%)
Other/Non-binary 6 (0.46%)

Sample 3 (MTurk convenience sample, NOT nationally representative), demographic breakdown:

Age N (%)
18 - 27 23 (27.06%)
28 - 37 37 (43.53%)
38 - 47 14 (16.47%)
48 - 57 6 (7.06%)
58+ 5 (5.88%)

Race N (%)
White 59 (69.41%)
Black/African-American 12 (14.12%)
Asian 5 (5.88%)
Hispanic/Latinx 4 (4.71%)
Multiracial 3 (3.53%)
Native American 2 (2.35%).

Gender N (%)
Male 46 (54.12%)
Female 38 (44.71%)
Other/Non-binary 1 (1.18%)

Sampling strategy	Sample 1: Nationally representative U.S. sample (age, race, gender). We used an online polling software (https://www.nbrii.com/our-process/sample-size-calculator/) to determine that at least 385 participants would be needed to obtain population estimates with a 5% margin of error and 95% confidence level. Anticipating participant exclusions, we over-sampled by about 15% and aimed to recruit 450 U.S. participants via the Prolific Academic platform (Prolific); 493 ultimately took the survey, each of whom was paid at a rate of $7.25 per hour. Seventy (70) participants were excluded based on the pre-registered exclusion criteria, leaving us with a final sample of 423 participants ("Sample 1") who completed an online survey.<br><br>Sample 2: U.S. convenience sample, data collected on Amazon's Mechanical Turk (MTurk). To power for the same confidence and margin of error as in Sample 1, but this time with a between-subjects design, it was determined that we would need ratings from 1,551 participants. Based on the Sample 1 exclusion rate, we over-recruited by about 10% and thus aimed to recruit 1,706 participants; 1,822 ultimately filled out at least part of the survey (not all finished), each of whom was paid $1.00. Five hundred and two (502) participants were excluded based on pre-registered exclusion criteria, leaving us with a final sample of 1,320 participants.<br><br>Sample 3. U.S. convenience sample, data collected on Amazon's Mechanical Turk (MTurk). We aimed to have as many observations per distribution as we had in Stage 1. For Stage 1, which was powered for a nationally representative sample across age, race, and gender, we recruited for 450 observations with a final sample of 423 (Sample 1). For the Sample 3 study, each participant would have to make three ratings for each measured construct rather than one (as in Stage 1), so we determined to recruit a third of the number of target participants as we had in our first sample to achieve similar statistical power. Accordingly, we recruited 150 participants, with 167 participants ultimately taking at least part of the survey (not all finished). Each participant who completed the survey was paid $1.00. Sixty-four participants (64) were excluded on the basis of the pre-registered exclusion criteria, leaving us with a final sample of N = 85.
Data collection	All studies conducted online using the Qualtrics survey platform, administered either through Prolific or MTurk. Because participants completed the surveys online (through the internet), there were no experimenters present; experimenters were thus, in effect, 'blind' to participant condition.
Timing	Sample 1: August 5-9, 2019<br>Sample 2: November 28 - December 11, 2019<br>Sample 3: April 27-28, 2020
Data exclusions	Sample 1: 70 participants were excluded based on preregistered exclusion criteria (see below).<br>Sample 2: 502 participants were excluded based on preregistered exclusion criteria (see below).<br>Sample 3: 64 participants were excluded [exploratory study; not preregistered] (see below).<br><br>Sample 1 exclusion details:<br>Did not reach main portion of survey (i.e., did not pass training) [Comprehension check]: 10<br>Failed text entry test [Bot check]: 6<br>Did not move slider to (at least) 1 of 2 specified positions [Attention check]: 54<br><br>Sample 2 exclusion details:<br>Failed Comprehension: 246<br>Did not move slider to (at least) 1 of 2 specified position [Attention check]: 171<br>Failed CAPTCHA test [Bot check]: 0<br>Failed text-entry test [Bot check]: 53<br>Being younger than 18 [Demographic check]: 2<br>Not fluent English speaker [Demographic check]: 12<br>Finished survey in< 4 min. [Quality check]: 73<br>Note: some participants met more than one criterion<br><br>Sample 3 exclusion details:<br>Did not move sliders to (at least) 1 of 2 specified positions [Attention check]: 64
Non-participation	Sample 1: 42 participants started but did not finish; 10 of those who did not finished were automatically "booted" upon failing the training/comprehension portion of the survey

	Sample 2: 128 participants started but did not finish (out of 1,822 who started)<br><br>Sample 3: 18 participants in the raw data file had no location information recorded and the data posted to Qualtrics nearly a week after the completion of the survey. We assumed these were bots and deleted those 18 lines of data prior to applying the exclusion criterion. Of the 149 remaining participants, all completed the survey.
Randomization	Samples 1 and 3 were within-subjects designs: all participants saw all conditions. Sample 2 was a between-subjects design: participants were randomly assigned to 1 of 13 conditions.

Reporting for specific materials, systems and methods

We require information from authors about some types of materials, experimental systems and methods used in many studies. Here, indicate whether each material, system or method listed is relevant to your study. If you are not sure if a list item applies to your research, read the appropriate section before selecting a response.

Materials & experimental systems

n/a	Involved in the study
☒	☐ Antibodies
☒	☐ Eukaryotic cell lines
☒	☐ Palaeontology and archaeology
☒	☐ Animals and other organisms
☐	☒ Human research participants
☒	☐ Clinical data
☒	☐ Dual use research of concern

Methods

n/a	Involved in the study
☒	☐ ChIP-seq
☒	☐ Flow cytometry
☒	☐ MRI-based neuroimaging

Human research participants

Policy information about studies involving human research participants

Population characteristics	We collected the following demographic data: gender, age, race, ethnicity, income, level of education, English fluency, political leanings on social and economic issues, religious affiliation and religiosity. Following best practices, due to the complex social implications of race and ethnicity, we did not include race or ethnicity as co-variates; nor did we include Fluency as a co-variate as non-Fluent speakers were excluded following our preregistration criteria. All other mentioned demographic information were used as co-variates.
Recruitment	Participants were recruited through Prolific (for the Sample 1 Nationally Representative Sample), and through Amazon's Mechanical Turk (using the TurkPrime interface) for Samples 2 and 3. Regarding potential biases in recruitment, the usual concerns about online recruitment of participants apply (e.g., only participants with access to a computer would be able to participate) and the MTurk samples, though more demographically diverse than traditional 'student samples' are not nationally representative. Finally, since we excluded participants who failed various attention checks, this may have affected the representativeness of the nationally representative Prolific sample. However, we don't see theoretical reasons why any of these considerations would substantively influence our results.
Ethics oversight	All studies were reviewed and approved by the Yale University Institutional review board (protocol #20000022385); informed consent was obtained from participants in each instance prior to data collection.

Note that full information on the approval of the study protocol must also be provided in the manuscript.
