# Supplementary material for: Author Correction: How social relationships shape moral wrongness judgments
Source: Nat Commun. 2024 Sep 10;15:7906. doi: 10.1038/s41467-024-52274-w (PMC11387412; doi:10.1038/s41467-024-52274-w)
Supplement: Supplementary file 3 — Replication Script [file 41467_2024_52274_MOESM3_ESM.zip › ReplicationScript/Reported Results.pdf]

Reported Results

September 18, 2022

1 Reported Results

This script reproduces the *updated* results reported in our manuscript, in the order in which they are reported. All linear mixed effects models from the main text are reproduced in the R script `Reported Results.R`.

1.1 Load Packages and Define Functions

[26]:
```
#Load required packages
import warnings
warnings.filterwarnings('ignore')
import pandas as pd
from scipy import stats
import statistics
import math
import numpy as np
import pingouin as pg
import statsmodels.api as sm
```

[27]:
```
#Function to calculate Cohen's d
def cohend(d1, d2):
    #Size of samples
    n1, n2 = len(d1), len(d2)
    #Variance of the samples
    s1, s2 = statistics.variance(d1), 0
    #Pooled standard deviation
    s = math.sqrt(((n1 - 1) * s1 + (n2 - 1) * s2) / (n1 + n2 - 2))
    #Means of the samples
    u1, u2 = np.mean(d1), np.mean(d2)
    #Effect size
    return (u1 - u2) / s
```

[28]:
```
#Function that creates a matrix containing the KS distance between each␣
 ↪relationship and itself and every other relationship
#for each function. The output is similar to a correlation matrix, but␣
 ↪populated with KS distance statistics.
def ks(df, scores, name, mean = True):
```

```python
#List of the functions
funcs = ['Care', 'Hierarchy', 'Mating', 'Reciprocity']
#List of the relationships
rels = ['Boss - Employee', 'Close Friends', 'Father - Child (under 18 y/o)',\
        'Friends-with-Benefits', 'Mother - Child (under 18 y/o)', 'Romantic Partners',\
        'Roommates / Housemates', 'Siblings', 'Strangers', 'Teammates']
#Empty list which will be populated with dataframes
frames = list()

#For each function
for func in funcs:
    #Create a dictionary with a key for each relationship
    data = {rel:[] for rel in rels}
    #For each relationship
    for rel in rels:
        #Get a list of functional expectation scores for the specific relationship and function
        rel_score = df[(df['relationship'] == rel) & (df['function'] == func)][scores].tolist()
        #For every relationship
        for other in rels:
            #Get a list of scores for the relationship and function
            other_score = df[(df['relationship'] == other) & (df['function'] == func)][scores].tolist()
            #Compute the KS distance between these scores and the scores in the outer loop
            D, p = stats.ks_2samp(rel_score, other_score)
            #Add the KS stat to the dictionary of relationships
            data[rel].append(D)
    #Create a dataframe from the relationship:stat pairs
    out_df = pd.DataFrame(data)
    #List of standardized relationship names
    viz_names = ['BE', 'CF', 'FC', 'FB', 'MC', 'RP', 'RH', 'SB', 'ST', 'TM']
    #Asign the relationship names to the columns and rows of the KS matrix/dataframe
    out_df.columns = viz_names
    out_df.index = viz_names
    #Add the dataframe to the list of dataframes
    frames.append(out_df)

if not mean:
        data2 = {'function':[], 'rel_1':[], 'rel_2':[], 'ks_' + name:[]}
        for i in range(len(frames)):
            for col in frames[i].columns:
```

```
                    for row in frames[i].index:
                        if col != row:
                            data2['function'].append(funcs[i])
                            data2['rel_1'].append(col)
                            data2['rel_2'].append(row)
                            data2['ks_' + name].append(frames[i].at[col, row])

            final_df = pd.DataFrame(data2)
            final_df.drop_duplicates(subset = ['function', 'rel_1', 'rel_2'],␣
↪inplace = True)
            final_df = final_df[~final_df[['function', 'rel_1', 'rel_2']].
↪apply(frozenset, axis=1).duplicated()]

    elif mean:
        data3 = {'rel_1':[], 'rel_2':[], 'ks_' + name + '_care':[], 'ks_' +␣
↪name + '_hier':[],
                 'ks_' + name + '_mate':[], 'ks_' + name + '_recip':[]}
        #Cycle through the columns and rows
        for col in frames[0].columns:
            for row in frames[0].index:
                if col != row:
                    #Populate a long-form dictionary of the relationship pairs␣
↪and their KS scores
                    data3['rel_1'].append(col)
                    data3['rel_2'].append(row)
                    data3['ks_' + name + '_care'].append(frames[0].at[col, row])
                    data3['ks_' + name + '_hier'].append(frames[1].at[col, row])
                    data3['ks_' + name + '_mate'].append(frames[2].at[col, row])
                    data3['ks_' + name + '_recip'].append(frames[3].at[col,␣
↪row])

        final_df = pd.DataFrame(data3)
        #Compute average metrics
        final_df['ks_' + name + '_mean'] = final_df[[col for col in final_df.
↪columns if 'ks' in col]].mean(axis = 1)
        final_df.drop(columns = ['ks_' + name + '_care', 'ks_' + name + '_hier',
                                 'ks_' + name + '_mate', 'ks_' + name +␣
↪'_recip'], inplace = True)

    return final_df
```

[29]:
```
#Function to calculate the difference between two strings (used for bot checks)
def distance(s, t):
    # Initialize matrix of zeros
    rows = len(s)+1
    cols = len(t)+1
```

```
    distance = np.zeros((rows,cols),dtype = int)
    # Populate matrix of zeros with the indeces of each character of both␣
↪strings
    for i in range(1, rows):
        for k in range(1,cols):
            distance[i][0] = i
            distance[0][k] = k
    # Iterate over the matrix to compute the cost of deletions,insertions and/
↪or substitutions
    for col in range(1, cols):
        for row in range(1, rows):
            if s[row-1] == t[col-1]:
                cost = 0 # If the characters are the same in the two strings in␣
↪a given position [i,j] then the cost is 0
            else:
                cost = 1
            distance[row][col] = min(distance[row-1][col] + 1,      # Cost of␣
↪deletions
                                 distance[row][col-1] + 1,          # Cost of␣
↪insertions
                                 distance[row-1][col-1] + cost)     # Cost of␣
↪substitutions

    # This is the minimum number of edits needed to convert string a to string b
    return distance[row][col]
```

[30]:

```
def gndr_diff(rel, func):
    dataf = s1[(s1["GenderMFO"] == 'Female') & (s1["relationship"] == rel) &␣
↪(s1["function"] == func)]['scaled_functional_score']
    datam = s1[(s1["GenderMFO"] == 'Male') & (s1["relationship"] == rel) &␣
↪(s1["function"] == func)]['scaled_functional_score']
    mf = dataf.mean()
    mm = datam.mean()
    mdiff = round(mf-mm, 2)
    return mdiff
```

1.2 Main Paper

1.3 Sample 1 Results

p. 3

[31]:

```
#Read in the data
s1 = pd.read_csv('sample1.csv')

#N
print("N =", len(s1["ResponseId"].unique()))
```

```
#Reciprocity
    #Mean and standard deviation
recipMean = s1[s1["function"] == "Reciprocity"]["raw_functional_score"].mean()
recipSd = s1[s1["function"] == "Reciprocity"]["raw_functional_score"].std()
print("\nReciprocity expectations")
print("\tMean = {:.2f}".format(recipMean))
print("\tSD = {:.2f}".format(recipSd), end = "\n\n")
    #Distance from scale midpoint of 0, and effect size measure
t, p = stats.ttest_1samp(s1[s1['function'] == "Reciprocity"]['raw_functional_score'].to_list(), 0)
d = cohend(s1[s1['function'] == "Reciprocity"]['raw_functional_score'].to_list(), [0])
print("\tTest if the mean is higher than the scale midpoint")
print("\tt({}) = {:.2f}".format(len(s1[s1['function'] == "Reciprocity"])-1, t), end = " ")
print("p = {:.4f}".format(p), end = " ")
print("d = {:.2f}".format(d))

#Mating
    #Mean and standard deviation
mateMean = s1[s1["function"] == "Mating"]["raw_functional_score"].mean()
mateSd = s1[s1["function"] == "Mating"]["raw_functional_score"].std()
print("\nMating expectations")
print("\tMean = {:.2f}".format(mateMean))
print("\tSD = {:.2f}".format(mateSd))
    #Distance from scale midpoint of 0
t, p = stats.ttest_1samp(s1[s1['function'] == "Mating"]['raw_functional_score'].to_list(), 0)
d = cohend(s1[s1['function'] == "Mating"]['raw_functional_score'].to_list(), [0])
print("\tTest if the mean is higher than the scale midpoint")
print("\tt({}) = {:.2f}".format(len(s1[s1['function'] == "Mating"])-1, t), end = " ")
print("p = {:.4f}".format(p), end = " ")
print("d = {:.2f}".format(d), end = "\n\n")
print("\t\tExceptions")
    #Exceptional relationships
romPar = s1[(s1['function'] == "Mating") & (s1["relationship"] == "Romantic Partners")]
romParMean = romPar["raw_functional_score"].mean()
romParSd = romPar["raw_functional_score"].std()
print("\n\t\tRomantic partners")
print("\t\t\tMean = {:.2f}".format(romParMean))
print("\t\t\tSD = {:.2f}".format(romParSd))
```

```
fwb = s1[(s1['function'] == "Mating") & (s1["relationship"] ==␣
 ↪"Friends-with-Benefits")]
fwbParMean = fwb["raw_functional_score"].mean()
fwbParSd = fwb["raw_functional_score"].std()
print("\n\t\tFriends-with-Benefits")
print("\t\t\tMean = {:.2f}".format(fwbParMean))
print("\t\t\tSD = {:.2f}".format(fwbParSd))

#Functional SDs of the means, across dyads
print("\nFunctional SDs of the means, across dyads")

grouped = pd.DataFrame(s1.groupby(["relationship",␣
 ↪"function"])["raw_functional_score"].std()).reset_index()

mate = grouped[grouped["function"] == "Mating"]
mateSdMeans = mate["raw_functional_score"].mean()
print("\tMating SD^mean = {:.2f}".format(mateSdMeans))

care = grouped[grouped["function"] == "Care"]
careSdMeans = care["raw_functional_score"].mean()
print("\tCare SD^mean = {:.2f}".format(careSdMeans))

recip = grouped[grouped["function"] == "Reciprocity"]
recipSdMeans = recip["raw_functional_score"].mean()
print("\tRecciprocity SD^mean = {:.2f}".format(recipSdMeans))

hier = grouped[grouped["function"] == "Hierarchy"]
hierSdMeans = hier["raw_functional_score"].mean()
print("\tHierarchy SD^mean = {:.2f}".format(hierSdMeans))

#Functionally "polarized" relationships
print('\nFunctionally "polarized" relationships / SD across functions')
grouped = pd.DataFrame(s1.groupby(["relationship",␣
 ↪"function"])["raw_functional_score"].mean()).reset_index()
grouped = grouped.pivot(index = "relationship", columns = "function").
 ↪reset_index()
grouped.columns = ["relationship", "care", "hier", "mate", "recip"]
grouped["sd"] = grouped[["care", "hier", "mate", "recip"]].std(axis = 1)

mcU18 = grouped[grouped["relationship"] == "Mother - Child (under 18 y/
 ↪o)"]["sd"].values[0]
fcU18 = grouped[grouped["relationship"] == "Father - Child (under 18 y/
 ↪o)"]["sd"].values[0]
strange = grouped[grouped["relationship"] == "Strangers"]["sd"].values[0]
print("\tMother - Child (under 18 y/o) = {:.2f}".format(mcU18))
print("\tFather - Child (under 18 y/o) = {:.2f}".format(fcU18))
```

```python
print("\tStrangers = {:.2f}".format(strange))

#Functionally "specific" relationships
print('\nFunctionally "specific" relationships')
groupedM = pd.DataFrame(s1.groupby(["relationship", "function"])["raw_functional_score"].mean()).reset_index()
roomHouseM = groupedM[groupedM["relationship"] == "Roommates / Housemates"]
bossEmployM = groupedM[groupedM["relationship"] == "Boss - Employee"]

groupedSd = pd.DataFrame(s1.groupby(["relationship", "function"])["raw_functional_score"].std(ddof = 0)).reset_index()
roomHouseSd = groupedSd[groupedSd["relationship"] == "Roommates / Housemates"]
bossEmploySd = groupedSd[groupedSd["relationship"] == "Boss - Employee"]

print("\tRoommates / Housemates")
careM = roomHouseM[roomHouseM["function"] == "Care"]["raw_functional_score"].values[0]
hierM = roomHouseM[roomHouseM["function"] == "Hierarchy"]["raw_functional_score"].values[0]
mateM = roomHouseM[roomHouseM["function"] == "Mating"]["raw_functional_score"].values[0]
recipM = roomHouseM[roomHouseM["function"] == "Reciprocity"]["raw_functional_score"].values[0]
careSd = roomHouseSd[roomHouseSd["function"] == "Care"]["raw_functional_score"].values[0]
hierSd = roomHouseSd[roomHouseSd["function"] == "Hierarchy"]["raw_functional_score"].values[0]
mateSd = roomHouseSd[roomHouseSd["function"] == "Mating"]["raw_functional_score"].values[0]
recipSd = roomHouseSd[roomHouseSd["function"] == "Reciprocity"]["raw_functional_score"].values[0]
print("\t\tCare Mean = {:.2f}".format(careM))
print("\t\tCare SD = {:.2f}".format(careSd))
print("\t\tHierarchy Mean = {:.2f}".format(hierM))
print("\t\tHierarchy SD = {:.2f}".format(hierSd))
print("\t\tMating Mean = {:.2f}".format(mateM))
print("\t\tMating SD = {:.2f}".format(mateSd))
print("\t\tReciprocity Mean = {:.2f}".format(recipM))
print("\t\tReciprocity SD = {:.2f}".format(recipSd))

print("\n\tBoss - Employee")
careM = bossEmployM[bossEmployM["function"] == "Care"]["raw_functional_score"].values[0]
hierM = bossEmployM[bossEmployM["function"] == "Hierarchy"]["raw_functional_score"].values[0]
```

```
mateM = bossEmployM[bossEmployM["function"] ==␣
  ↪"Mating"]["raw_functional_score"].values[0]
recipM = bossEmployM[bossEmployM["function"] ==␣
  ↪"Reciprocity"]["raw_functional_score"].values[0]
careSd = bossEmploySd[bossEmploySd["function"] ==␣
  ↪"Care"]["raw_functional_score"].values[0]
hierSd = bossEmploySd[bossEmploySd["function"] ==␣
  ↪"Hierarchy"]["raw_functional_score"].values[0]
mateSd = bossEmploySd[bossEmploySd["function"] ==␣
  ↪"Mating"]["raw_functional_score"].values[0]
recipSd = bossEmploySd[bossEmploySd["function"] ==␣
  ↪"Reciprocity"]["raw_functional_score"].values[0]
print("\t\tCare Mean = {:.2f}".format(careM))
print("\t\tCare SD = {:.2f}".format(careSd))
print("\t\tHierarchy Mean = {:.2f}".format(hierM))
print("\t\tHierarchy SD = {:.2f}".format(hierSd))
print("\t\tMating Mean = {:.2f}".format(mateM))
print("\t\tMating SD = {:.2f}".format(mateSd))
print("\t\tReciprocity Mean = {:.2f}".format(recipM))
print("\t\tReciprocity SD = {:.2f}".format(recipSd))

#Functionally "pluralistic" relationship
print('\nFunctionally "pluralistic" relationship')
grouped = pd.DataFrame(s1.groupby(["relationship"])["raw_functional_score"].
  ↪mean()).reset_index()
print("\tRomantic partners")
romPar = grouped[grouped["relationship"] == "Romantic␣
  ↪Partners"]["raw_functional_score"].values[0]
print("\t\tMean across functions = {:.2f}".format(romPar))
grouped = pd.DataFrame(s1.groupby(["relationship",␣
  ↪"function"])["raw_functional_score"].std(ddof = 0)).reset_index()

groupedM = pd.DataFrame(s1.groupby(['relationship',␣
  ↪"function"])["raw_functional_score"].mean()).reset_index()
groupedM = groupedM[groupedM["relationship"] == "Romantic Partners"]
groupedSd = pd.DataFrame(s1.groupby(['relationship',␣
  ↪"function"])["raw_functional_score"].std(ddof = 0)).reset_index()
groupedSd = groupedSd[groupedSd["relationship"] == "Romantic Partners"]
print("\t\tCare")
print("\t\t\tMean = {:.2f}".format(groupedM[groupedM["function"] ==␣
  ↪"Care"]["raw_functional_score"].values[0]))
print("\t\t\tSD = {:.2f}".format(groupedSd[groupedSd["function"] ==␣
  ↪"Care"]["raw_functional_score"].values[0]))

print("\t\tMating")
```

```python
print("\t\t\tMean = {:.2f}".format(groupedM[groupedM["function"] ==␣
↪"Mating"]["raw_functional_score"].values[0]))
print("\t\t\tSD = {:.2f}".format(groupedSd[groupedSd["function"] ==␣
↪"Mating"]["raw_functional_score"].values[0]))

print("\n\t\tReciprocity")
print("\t\t\tMean = {:.2f}".format(groupedM[groupedM["function"] ==␣
↪"Reciprocity"]["raw_functional_score"].values[0]))
print("\t\t\tSD = {:.2f}".format(groupedSd[groupedSd["function"] ==␣
↪"Reciprocity"]["raw_functional_score"].values[0]))
```

```
N = 423

Reciprocity expectations
        Mean = 54.23
        SD = 49.64

        Test if the mean is higher than the scale midpoint
        t(8459) = 100.47 p = 0.0000 d = 1.09

Mating expectations
        Mean = -63.02
        SD = 62.01
        Test if the mean is higher than the scale midpoint
        t(8459) = -93.48 p = 0.0000 d = -1.02

                Exceptions

                Romantic partners
                        Mean = 95.12
                        SD = 12.94

                Friends-with-Benefits
                        Mean = 58.43
                        SD = 51.21

Functional SDs of the means, across dyads
        Mating SD^mean = 32.26
        Care SD^mean = 37.82
        Reccíprocity SD^mean = 42.25
        Hierarchy SD^mean = 53.72

Functionally "polarized" relationships / SD across functions
        Mother - Child (under 18 y/o) = 85.33
        Father - Child (under 18 y/o) = 85.20
        Strangers = 37.93
```

```
Functionally "specific" relationships
        Roommates / Housemates
                Care Mean = 24.90
                Care SD = 43.64
                Hierarchy Mean = -4.48
                Hierarchy SD = 63.00
                Mating Mean = -52.39
                Mating SD = 49.85
                Reciprocity Mean = 87.30
                Reciprocity SD = 21.71

        Boss - Employee
                Care Mean = 7.86
                Care SD = 50.21
                Hierarchy Mean = 84.75
                Hierarchy SD = 24.68
                Mating Mean = -92.17
                Mating SD = 23.98
                Reciprocity Mean = 29.14
                Reciprocity SD = 58.93

Functionally "pluralistic" relationship
        Romantic partners
                Mean across functions = 64.61
                Care
                        Mean = 92.43
                        SD = 17.06
                Mating
                        Mean = 95.12
                        SD = 12.92

                Reciprocity
                        Mean = 84.95
                        SD = 27.28
```

Gender differences in relational norm expectations*pg.3*

[32]:
```
dataf = s1[(s1["GenderMFO"] == "Female") &
           (s1["function"] == "Care")]['scaled_functional_score']
mf = round(dataf.mean(), 2)
sdf = round(dataf.std(), 2)


datam = s1[(s1["GenderMFO"] == "Male") &
           (s1["function"] == "Care")]['scaled_functional_score']
mm = round(datam.mean(), 2)
sdm = round(datam.std(), 2)
```

```
print("Gendered expectations for care:")
print("\tWomen: M = {}, SD = {}".format(mf, sdf))
print("\tMen: M = {}, SD = {}".format(mm, sdm))
```

```
Gendered expectations for care:
        Women: M = 0.43, SD = 0.75
        Men: M = 0.37, SD = 0.79
```

[33]:
```
print("Relationships with the greatest gender difference for care expectations␣
 ↪(Female - Male):")
print("\tRoommates / Housemates")
mdiff = gndr_diff("Roommates / Housemates", "Care")
print("\t\t{}".format(mdiff))
print("\tCustomer - Seller")
mdiff = gndr_diff("Customer - Seller", "Care")
print("\t\t{}".format(mdiff))
print("\tTeacher - Student")
mdiff = gndr_diff("Teacher - Student", "Care")
print("\t\t{}".format(mdiff))
print("\tNeighbors")
mdiff = gndr_diff("Neighbors", "Care")
print("\t\t{}".format(mdiff))
print("\tColleagues / Classmates")
mdiff = gndr_diff("Colleagues / Classmates", "Care")
print("\t\t{}".format(mdiff))
```

```
Relationships with the greatest gender difference for care expectations (Female
- Male):
        Roommates / Housemates
                0.17
        Customer - Seller
                0.15
        Teacher - Student
                0.14
        Neighbors
                0.14
        Colleagues / Classmates
                0.13
```

[34]:
```
dataf = s1[(s1["GenderMFO"] == "Female") &
           (s1["function"] == "Mating")]['scaled_functional_score']
mf = round(dataf.mean(), 2)
sdf = round(dataf.std(), 2)


datam = s1[(s1["GenderMFO"] == "Male") &
```

```python
            (s1["function"] == "Mating")]['scaled_functional_score']
mm = round(datam.mean(), 2)
sdm = round(datam.std(), 2)

print("Gendered expectations for mating:")
print("\tWomen: M = {}, SD = {}".format(mf, sdf))
print("\tMen: M = {}, SD = {}".format(mm, sdm))
```

```
Gendered expectations for mating:
        Women: M = -1.19, SD = 0.89
        Men: M = -1.12, SD = 0.97
```

[35]:

```python
print("Relationships with the greatest gender difference for mating␣
 ↪expectations (Male - Female):")
print("\tFriends-with-Benefits")
mdiff = gndr_diff("Friends-with-Benefits", "Mating")
print("\t\t{}".format(abs(mdiff)))
print("\tRoommates / Housemates")
mdiff = gndr_diff("Roommates / Housemates", "Mating")
print("\t\t{}".format(abs(mdiff)))
print("\tAcquaintances")
mdiff = gndr_diff("Acquaintances", "Mating")
print("\t\t{}".format(abs(mdiff)))
print("\tClose Friends")
mdiff = gndr_diff("Close Friends", "Mating")
print("\t\t{}".format(abs(mdiff)))
print("\tColleagues / Classmates")
mdiff = gndr_diff("Colleagues / Classmates", "Mating")
print("\t\t{}".format(abs(mdiff)))
print("\tStrangers")
mdiff = gndr_diff("Strangers", "Mating")
print("\t\t{}".format(abs(mdiff)))
print("\tNeighbors")
mdiff = gndr_diff("Neighbors", "Mating")
print("\t\t{}".format(abs(mdiff)))
```

```
Relationships with the greatest gender difference for mating expectations (Male
- Female):
        Friends-with-Benefits
                0.27
        Roommates / Housemates
                0.25
        Acquaintances
                0.24
        Close Friends
                0.25
```

```
        Colleagues / Classmates
                0.2
        Strangers
                0.16
        Neighbors
                0.17
```

1.4 Moral Judgment Ratings and Sample 2

p.5

[36]:
```
#Read in the ratings of the moral action statements
ratings = pd.read_csv("action_ratings.csv")
print("Moral action statement ratings:")
#Make a longform copy of the data
icc_df = pd.melt(ratings, id_vars = "rater", var_name = "item", value_name =␣
 ↪'rating')
#Run the ICC
icc = pg.intraclass_corr(data = icc_df, targets = 'item', raters = 'rater',␣
 ↪ratings = 'rating')
print("\tICC =", round(icc["ICC"][5], 2), end = "\n\n")

cryCareM = round(ratings["C-CryingWalk-C-W - Care function"].mean(), 2)
cryCareSD = round(ratings["C-CryingWalk-C-W - Care function"].std(), 2)
print("\tCrying and walking away\n\t\tEffect on the care function")
print("\t\t\tMean =", cryCareM)
print("\t\t\tSD =", cryCareSD)

cryMateM = round(ratings["M-CryingWalk-C-W - Mating function"].mean(), 2)
cryMateSD = round(ratings["M-CryingWalk-C-W - Mating function"].std(), 2)
print("\n\t\tEffect on the mating function")
print("\t\t\tMean =", cryMateM)
print("\t\t\tSD =", cryMateSD, end = "\n\n")


#Read in the sample 2 data
s2 = pd.read_csv("sample2.csv")

#N
print('Sample 2:')
print("\tN = {:.0f}".format(len(s2["ResponseId"].unique())))
```

```
Moral action statement ratings:
        ICC = 0.97

        Crying and walking away
                Effect on the care function
                        Mean = -87.93
```

```
                SD = 15.46

        Effect on the mating function
                Mean = -40.13
                SD = 35.02

Sample 2:
        N = 1320
```

1.5 Main hypotheses

p.6

[37]:
```python
#LME models and results are presented in the script `Reported Results.R`
```

1.6 Correlation analysis

p.7 Correlating the distance between each dyad in moral judgment space with that in relational norm space

[38]:
```python
#Calculate KS distance for the moral wrongness ratings and again for the␣
↪functional expectations
funcKs = ks(s1, 'scaled_functional_score', 'func')
wrongKs = ks(s2, 'wrongness', 'wrong')
#Merge the wrongness and norm distances into a single dataframe
m = wrongKs.set_index(['rel_1', 'rel_2']).join(funcKs.set_index(['rel_1',␣
↪'rel_2'])).reset_index()
m = m[~m[['rel_1', 'rel_2']].apply(frozenset, axis = 1).duplicated()]
m.reset_index(inplace = True, drop = True)
```

[39]:
```python
#Compute the correlations
print('Overall KS correlation:')
r, p = stats.spearmanr(m['ks_func_mean'], m['ks_wrong_mean'])
print("\tr = {:.2f}".format(r), end = " ")
print("\tp = {:.4f}".format(p), end = "\n\n")

#Run the same analyses, but for each function
func = ks(s1, 'scaled_functional_score', 'func', mean = False)
wrong = ks(s2, 'wrongness', 'wrong', mean = False)
ks = func.set_index(['function', 'rel_1', 'rel_2']).join(
    wrong.set_index(['function', 'rel_1', 'rel_2']), how = 'left')
ks.reset_index(inplace = True)
ks = ks[~ks[['function', 'rel_1', 'rel_2']].apply(frozenset, axis=1).
↪duplicated()]
ks.reset_index(drop = True, inplace = True)

care = ks[ks['function'] == 'Care']
hier = ks[ks['function'] == 'Hierarchy']
```

```
mate = ks[ks['function'] == 'Mating']
recip = ks[ks['function'] == 'Reciprocity']

print('KS correlations for each function:')
print('\tCare')
r, p = stats.spearmanr(care['ks_func'], care['ks_wrong'])
print("\t\tr = {:.2f}".format(r), end = " ")
print("\t\tp = {:.4f}".format(p), end = "\n\n")
print('\tHierarchy')
r, p = stats.spearmanr(hier['ks_func'], hier['ks_wrong'])
print("\t\tr = {:.2f}".format(r), end = " ")
print("\t\tp = {:.2f}".format(p), end = "\n\n")
print('\tMating')
r, p = stats.spearmanr(mate['ks_func'], mate['ks_wrong'])
print("\t\tr = {:.2f}".format(r), end = " ")
print("\t\tp = {:.4f}".format(p), end = "\n\n")
print('\tReciprocity')
r, p = stats.spearmanr(recip['ks_func'], recip['ks_wrong'])
print("\t\tr = {:.2f}".format(r), end = " ")
print("\t\tp = {:.2f}".format(p), end = "\n\n")

print("Sample 3:")
s3 = pd.read_csv("sample3.csv")
print("\tN = ", len(s3))
```

```
Overall KS correlation:
        r = 0.43        p = 0.0029

KS correlations for each function:
        Care
                r = 0.50                p = 0.0005

        Hierarchy
                r = 0.29                p = 0.05

        Mating
                r = 0.69                p = 0.0000

        Reciprocity
                r = -0.10               p = 0.49

Sample 3:
        N =  85
```

1.7 Alternative models

p.9

[40]:
```
#LME models and results are presented in the script `Reported Results.R`
```

2 Supplementary Materials

2.1 Stage 1

1.1.

[41]:
```
#Load the sample 1 raw dataset
s1r = pd.read_csv("sample1_raw.csv")
#Reduce to columns of interest
keep = ['ResponseId'] + [col for col in s1r if col.startswith('I.')] +␣
 ↪['Q349_1', 'GenderMFO', 'Age', 'Ethnicity',
                                                                     ␣
 ↪'Fluency', 'Income', 'Education',
                                                                     ␣
 ↪'Political - Social_1',
                                                                     ␣
 ↪'Political - Economic_1',
                                                                      'How.
 ↪Religious_1', 'BotCheckFRIDAY',
                                                                     ␣
 ↪'Q303', 'Q350_1']
s1r = s1r[keep]
#Handle column type
num = [col for col in s1r if col.startswith('I.')] + ['Q349_1']
s1r[num] = s1r[num].apply(pd.to_numeric)
#Fill missing values with the column means
score_cols = [col for col in s1r if col.startswith('I.')]
s1r[score_cols] = s1r[score_cols].apply(lambda x: x.fillna(x.mean()), axis = 0)
pre_excl = s1r.copy()
#Run exclusions
print('Pre-exclusions:\t\t' + str(len(s1r)))
s1r = s1r[s1r['Q303'] != 'I understand.']
print('Pre-training:\t\t' + str(len(s1r)))
s1r = s1r[s1r['Q349_1'] >= 95]
s1r = s1r[s1r['Q350_1'] <= -95]
print('Post-attention check:\t' + str(len(s1r)))
#Lowercase the botcheck and remove whitespace
s1r['BotCheckFRIDAY'] = s1r['BotCheckFRIDAY'].str.lower()
s1r['BotCheckFRIDAY'] = s1r['BotCheckFRIDAY'].str.strip()
s1r['BotCheckFRIDAY'] = s1r['BotCheckFRIDAY'].str.replace(" ","")
#Calculates distance and edits from the correct answer
s1r = s1r[s1r['BotCheckFRIDAY'].notna()]
s1r['bot_dist'] = s1r['BotCheckFRIDAY'].apply(lambda x: distance(x, 'friday'))
s1r = s1r[s1r['bot_dist'] <= 1]
print('Post-bot check:\t\t' + str(len(s1r)))
```

```
s1r.drop(columns = ['bot_dist', 'BotCheckFRIDAY', 'Q349_1', 'Q350_1', 'Q303'],␣
↪inplace = True)
```

```
Pre-exclusions:         493
Pre-training:           483
Post-attention check:   429
Post-bot check:         423
```

[42]:
```
s1r["GenderMFO"].value_counts()
```

[42]:
```
Female    217
Male      201
Other       4
Name: GenderMFO, dtype: int64
```

[43]:
```
ageM = round(s1r["Age"].mean(), 2)
ageSD = round(s1r["Age"].std(), 2)
ageMin = s1r["Age"].min()
ageMax = s1r["Age"].max()
print("Ages {} to {}, Mean = {}, SD = {}".format(ageMin, ageMax, ageM, ageSD))
```

```
Ages 18.0 to 79.0, Mean = 44.25, SD = 15.67
```

[44]:
```
conditions = [
    s1r["Age"].isin(range(18,28)),
    s1r["Age"].isin(range(28,38)),
    s1r["Age"].isin(range(38,48)),
    s1r["Age"].isin(range(48,58)),
    s1r["Age"] >= 58,
    s1r["Age"].isna(),
]

choices = ["18-27", "28-37", "38-47", "48-57", "58+", "Missing"]

s1r["age_brackets"] = np.select(conditions, choices)
age = pd.DataFrame(s1r["age_brackets"].value_counts())
age["Sample%"] = round((age["age_brackets"]/len(s1r)) * 100, 2)
age
```

[44]:

	age_brackets	Sample%
58+	117	27.66
18-27	82	19.39
28-37	78	18.44
48-57	75	17.73
38-47	70	16.55
Missing	1	0.24

[45]:
```python
s1r["Ethnicity"] = np.where(s1r["Ethnicity"].isna(), "Missing",␣
↪s1r["Ethnicity"])
race = pd.DataFrame(s1r["Ethnicity"].value_counts())
race["Sample%"] = round((race["Ethnicity"]/len(s1r)) * 100, 2)
race
```

[45]:

	Ethnicity	Sample%
White	296	69.98
Black/African American	60	14.18
Asian	29	6.86
Hispanic/Latinx	21	4.96
Other	10	2.36
American Indian/Alaska Native	4	0.95
Hawaiian/Pacific Islander	2	0.47
Missing	1	0.24

[46]:
```python
s1r["GenderMFO"] = np.where(s1r["GenderMFO"].isna(), "Missing",␣
↪s1r["GenderMFO"])
gndr = pd.DataFrame(s1r["GenderMFO"].value_counts())
gndr["Sample%"] = round((gndr["GenderMFO"]/len(s1r)) * 100, 2)
gndr
```

[46]:

	GenderMFO	Sample%
Female	217	51.30
Male	201	47.52
Other	4	0.95
Missing	1	0.24

1.4

[47]:
```python
func_var = pd.DataFrame(s1.groupby(['function',␣
↪'relationship'])["raw_functional_score"].mean()).reset_index()
func_var = func_var.pivot(index = "relationship", columns = 'function', values␣
↪= 'raw_functional_score')
func_var['SD'] = func_var[['Care', "Hierarchy", 'Mating', 'Reciprocity']].
↪std(axis = 1)
func_var.round(2).sort_values(by = "SD", ascending = False)
```

[47]:

function	Care	Hierarchy	Mating	Reciprocity	SD
relationship					
Mother - Child (under 18 y/o)	95.53	65.39	-98.22	9.05	85.33
Father - Child (under 18 y/o)	93.99	67.39	-98.37	12.93	85.20
Siblings	84.06	-0.83	-98.47	74.96	84.64
Father - Child (over 18 y/o)	86.58	35.97	-97.59	53.62	80.92
Mother - Child (over 18 y/o)	88.05	28.02	-97.69	49.75	80.41
Extended Family Members	64.65	15.22	-96.40	57.26	74.32
Teacher - Student	42.97	72.77	-95.61	24.49	73.90

Boss - Employee	7.86	84.75	-92.17	29.14	73.87
Doctor - Patient	53.75	41.63	-95.31	30.40	69.28
Close Friends	79.39	-20.32	-50.31	79.96	67.51
Teammates	50.53	31.86	-73.00	75.43	65.29
Customer - Seller	-18.37	22.04	-81.77	60.39	60.77
Roommates / Housemates	24.90	-4.48	-52.39	87.30	58.42
Political Party Members	7.60	45.24	-66.04	63.82	57.44
Colleagues / Classmates	17.38	18.92	-60.00	77.29	56.32
Neighbors	13.08	-16.25	-57.99	67.57	52.77
Romantic Partners	92.43	-14.07	95.12	84.95	52.63
Friends-with-Benefits	28.13	-30.36	58.43	59.87	42.21
Acquaintances	-2.74	-3.54	-46.82	51.38	40.21
Strangers	-26.62	-11.00	-55.87	34.95	37.93

[48]:
```python
func_specific = func_var.copy()
func_specific['sum'] = func_specific[['Care', 'Hierarchy', 'Mating',␣
↪'Reciprocity']].sum(axis = 1)
func_specific['func_mean'] = func_specific[['Care', 'Hierarchy', 'Mating',␣
↪'Reciprocity']].mean(axis = 1)
func_specific['max'] = func_specific[['Care', 'Hierarchy', 'Mating',␣
↪'Reciprocity']].max(axis = 1)
func_specific['other_sum'] = func_specific['sum'] - func_specific['max']
func_specific['max_other_diff'] = func_specific['max'] -␣
↪func_specific['other_sum']
func_specific = func_specific.drop(columns = ["SD", "sum", "func_mean"])
func_specific['highest_M_function'] = func_specific[["Care", "Hierarchy",␣
↪"Mating", "Reciprocity"]].idxmax(axis = 1)
func_specific.round(2).sort_values(by = 'max_other_diff', ascending = False)
```

[48]:

function	Care	Hierarchy	Mating	Reciprocity	max \
relationship					
Boss - Employee	7.86	84.75	-92.17	29.14	84.75
Customer - Seller	-18.37	22.04	-81.77	60.39	60.39
Neighbors	13.08	-16.25	-57.99	67.57	67.57
Strangers	-26.62	-11.00	-55.87	34.95	34.95
Mother - Child (under 18 y/o)	95.53	65.39	-98.22	9.05	95.53
Roommates / Housemates	24.90	-4.48	-52.39	87.30	87.30
Father - Child (under 18 y/o)	93.99	67.39	-98.37	12.93	93.99
Siblings	84.06	-0.83	-98.47	74.96	84.06
Mother - Child (over 18 y/o)	88.05	28.02	-97.69	49.75	88.05
Acquaintances	-2.74	-3.54	-46.82	51.38	51.38
Colleagues / Classmates	17.38	18.92	-60.00	77.29	77.29
Teacher - Student	42.97	72.77	-95.61	24.49	72.77
Father - Child (over 18 y/o)	86.58	35.97	-97.59	53.62	86.58
Extended Family Members	64.65	15.22	-96.40	57.26	64.65
Doctor - Patient	53.75	41.63	-95.31	30.40	53.75
Political Party Members	7.60	45.24	-66.04	63.82	63.82

```
Close Friends                  79.39     -20.32  -50.31         79.96  79.96
Teammates                      50.53      31.86  -73.00         75.43  75.43
Friends-with-Benefits          28.13     -30.36   58.43         59.87  59.87
Romantic Partners              92.43     -14.07   95.12         84.95  95.12

function                       other_sum  max_other_diff highest_M_function
relationship
Boss - Employee                   -55.17          139.92          Hierarchy
Customer - Seller                 -78.09          138.49        Reciprocity
Neighbors                         -61.15          128.72        Reciprocity
Strangers                         -93.49          128.44        Reciprocity
Mother - Child (under 18 y/o)     -23.78          119.31               Care
Roommates / Housemates            -31.96          119.25        Reciprocity
Father - Child (under 18 y/o)     -18.05          112.04               Care
Siblings                          -24.34          108.40               Care
Mother - Child (over 18 y/o)      -19.91          107.96               Care
Acquaintances                     -53.09          104.47        Reciprocity
Colleagues / Classmates           -23.70          100.99        Reciprocity
Teacher - Student                 -28.14          100.91          Hierarchy
Father - Child (over 18 y/o)       -8.00           94.59               Care
Extended Family Members           -23.92           88.57               Care
Doctor - Patient                  -23.29           77.04               Care
Political Party Members           -13.20           77.02        Reciprocity
Close Friends                       8.77           71.19        Reciprocity
Teammates                           9.38           66.05        Reciprocity
Friends-with-Benefits              56.20            3.68        Reciprocity
Romantic Partners                 163.31          -68.18             Mating
```

[49]:
```
s1_mod_data = s1[s1['GenderMFO'] != 'Other']
s1_mod_data['GenderMFO'] = np.where(s1_mod_data['GenderMFO'] == 'Male', 0, 1)
s1_mod_data['Ethnicity'] = np.where(s1_mod_data['Ethnicity'] == 'White', 0, 1)
s1_mod_data['Income'] = np.where(s1_mod_data['Income'] <= 4, 0, 1)

m_econ = s1_mod_data['Political - Economic_1'].mean()
m_soc = s1_mod_data['Political - Social_1'].mean()
m_relig = s1_mod_data['how_religious'].mean()
s1_mod_data['poli_econ'] = np.where(s1_mod_data['Political - Economic_1'] <= m_econ, 0, 1)
s1_mod_data['poli_social'] = np.where(s1_mod_data['Political - Social_1'] <= m_soc, 0, 1)
s1_mod_data['how_religious'] = np.where(s1_mod_data['how_religious'] <= m_relig, 0, 1)
s1_mod_data = s1_mod_data.dropna()
```

[50]:
```
fml = 'raw_functional_score ~ C(GenderMFO) + C(Income) + C(how_religious) + C(poli_social) + C(poli_econ)'
vcf = {'relationship': '0 + C(relationship)'}
```

```
model = sm.MixedLM.from_formula(
    fml,
    vc_formula = vcf,
    groups = 'ResponseId',
    data = s1_mod_data)
result = model.fit(method='powell')

print(result.summary())
var_resid = result.scale
var_random_effect = float(result.summary().tables[1].iloc[-1][0])
var_fixed_effect = result.predict(s1_mod_data).var()
total_var = var_fixed_effect + var_random_effect + var_resid
marginal_r2 = var_fixed_effect / total_var
conditional_r2 = (var_fixed_effect + var_random_effect) / total_var
print('Conditional R2 = {}'.format(round(conditional_r2, 5)))
print("Converged:\t{}".format(result.converged))
```

```
              Mixed Linear Model Regression Results
==================================================================
Model:              MixedLM Dependent Variable: raw_functional_score
No. Observations:   33360   Method:             REML
No. Groups:         417     Scale:              5516.8509
Min. group size:    80      Log-Likelihood:     -191036.3611
Max. group size:    80      Converged:          Yes
Mean group size:    80.0
------------------------------------------------------------------
                        Coef.  Std.Err.   z    P>|z| [0.025 0.975]
------------------------------------------------------------------
Intercept               13.744    0.995 13.819 0.000 11.795 15.693
C(GenderMFO)[T.1]        0.995    0.843  1.181 0.238 -0.657  2.647
C(Income)[T.1]          -1.170    0.824 -1.420 0.156 -2.786  0.445
C(how_religious)[T.1]    0.607    0.884  0.687 0.492 -1.125  2.338
C(poli_social)[T.1]      2.493    1.136  2.195 0.028  0.267  4.718
C(poli_econ)[T.1]       -2.099    1.124 -1.868 0.062 -4.301  0.103
relationship Var         0.000    0.846
==================================================================

Conditional R2 = 0.00028
Converged:      True
```

[51]:
```
functions = ['Care', 'Hierarchy', 'Mating', 'Reciprocity']
for i in range(0, len(functions)):
    cur_df = s1_mod_data[s1_mod_data['function'] == functions[i]]
    fml = 'raw_functional_score ~ C(GenderMFO) + C(Income) + C(how_religious) +␣
 ↪C(poli_social) + C(poli_econ)'
    vcf = {'relationship': '0 + C(relationship)'}
    model = sm.MixedLM.from_formula(
```

```
        fml,
        vc_formula = vcf,
        groups = 'ResponseId',
        data = cur_df)
    result = model.fit(method='powell')

    print('*'*75)
    print(functions[i])
    print('*'*75)
    print(result.summary())
    var_resid = result.scale
    var_random_effect = float(result.summary().tables[1].iloc[-1][0])
    var_fixed_effect = result.predict(cur_df).var()
    total_var = var_fixed_effect + var_random_effect + var_resid
    marginal_r2 = var_fixed_effect / total_var
    conditional_r2 = (var_fixed_effect + var_random_effect) / total_var
    print('Conditional R2 = {}'.format(round(conditional_r2, 5)))
    print("Converged:\t{}".format(result.converged))
```

```
***************************************************************************
Care
***************************************************************************
              Mixed Linear Model Regression Results
==================================================================
Model:             MixedLM Dependent Variable: raw_functional_score
No. Observations:  8340    Method:             REML
No. Groups:        417     Scale:              621.0645
Min. group size:   20      Log-Likelihood:     -45407.5671
Max. group size:   20      Converged:          Yes
Mean group size:   20.0
------------------------------------------------------------------
                       Coef.   Std.Err.   z    P>|z| [0.025 0.975]
------------------------------------------------------------------
Intercept               39.838    1.502 26.527 0.000 36.894 42.781
C(GenderMFO)[T.1]        6.363    1.273  5.000 0.000  3.869  8.858
C(Income)[T.1]          -0.566    1.244 -0.455 0.649 -3.005  1.873
C(how_religious)[T.1]    5.045    1.334  3.781 0.000  2.430  7.660
C(poli_social)[T.1]      0.644    1.715  0.375 0.707 -2.717  4.004
C(poli_econ)[T.1]       -2.103    1.697 -1.240 0.215 -5.428  1.222
relationship Var      2523.724
==================================================================

Conditional R2 = 0.80376
Converged:      True
***************************************************************************
Hierarchy
***************************************************************************
```

```
              Mixed Linear Model Regression Results
======================================================================
Model:              MixedLM  Dependent Variable:  raw_functional_score
No. Observations:   8340     Method:              REML
No. Groups:         417      Scale:               822.5610
Min. group size:    20       Log-Likelihood:      -46492.4312
Max. group size:    20       Converged:           Yes
Mean group size:    20.0
----------------------------------------------------------------------
                       Coef.     Std.Err.     z    P>|z| [0.025 0.975]
----------------------------------------------------------------------
Intercept               21.457        1.711 12.544 0.000 18.104 24.809
C(GenderMFO)[T.1]       -0.039        1.449 -0.027 0.979 -2.880  2.802
C(Income)[T.1]           1.463        1.417  1.032 0.302 -1.315  4.241
C(how_religious)[T.1]   -1.266        1.520 -0.833 0.405 -4.244  1.713
C(poli_social)[T.1]      1.478        1.953  0.757 0.449 -2.350  5.306
C(poli_econ)[T.1]       -1.648        1.932 -0.853 0.394 -5.435  2.140
relationship Var      3257.424 38424018.981
======================================================================

Conditional R2 = 0.79846
Converged:      True
***************************************************************************
Mating
***************************************************************************
              Mixed Linear Model Regression Results
=====================================================================
Model:             MixedLM  Dependent Variable:  raw_functional_score
No. Observations:  8340     Method:              REML
No. Groups:        417      Scale:               764.6697
Min. group size:   20       Log-Likelihood:      -46221.7151
Max. group size:   20       Converged:           Yes
Mean group size:   20.0
---------------------------------------------------------------------
                       Coef.   Std.Err.    z    P>|z|  [0.025  0.975]
---------------------------------------------------------------------
Intercept             -57.420    1.656 -34.676 0.000 -60.665 -54.174
C(GenderMFO)[T.1]      -7.146    1.403  -5.093 0.000  -9.896  -4.396
C(Income)[T.1]         -3.609    1.372  -2.630 0.009  -6.298  -0.920
C(how_religious)[T.1]  -2.993    1.471  -2.034 0.042  -5.876  -0.109
C(poli_social)[T.1]     7.227    1.891   3.823 0.000   3.522  10.933
C(poli_econ)[T.1]      -5.654    1.871  -3.022 0.003  -9.320  -1.988
relationship Var     3058.679
=====================================================================

Conditional R2 = 0.80132
Converged:      True
***************************************************************************
```

```
Reciprocity
***************************************************************************
              Mixed Linear Model Regression Results
==================================================================
Model:              MixedLM Dependent Variable: raw_functional_score
No. Observations: 8340    Method:             REML
No. Groups:         417     Scale:              493.6351
Min. group size:  20      Log-Likelihood:     -44398.0382
Max. group size:  20      Converged:          Yes
Mean group size:  20.0
------------------------------------------------------------------
                       Coef.   Std.Err.   z    P>|z| [0.025 0.975]
------------------------------------------------------------------
Intercept                51.101    1.330 38.409 0.000 48.493 53.709
C(GenderMFO)[T.1]         4.801    1.127  4.259 0.000  2.592  7.011
C(Income)[T.1]           -1.970    1.102 -1.787 0.074 -4.130  0.191
C(how_religious)[T.1]     1.641    1.182  1.388 0.165 -0.676  3.957
C(poli_social)[T.1]       0.622    1.519  0.409 0.682 -2.355  3.599
C(poli_econ)[T.1]         1.008    1.503  0.671 0.502 -1.937  3.954
relationship Var       1974.540
==================================================================

Conditional R2 = 0.80075
Converged:      True
```

2.2 Stage 2

2.2

[52]:

```
s2r = pd.read_csv('sample2_raw.csv', lineterminator = '\n')
#Clean, type-control, fill missing values, and reduce to columns of interest
cond = ['ResponseId', 'Condition']
scores = [col for col in s2r.columns if col.startswith('M.') or col.
 ↪startswith('L.')]
attn = ['Duration (in seconds)', 'CheckNo1Wrong', 'CheckNo2Wrong',␣
 ↪'Check100_1', 'Check0_1', 'BotCheckSET']
demos = ['Gender', 'Age', 'Ethnicity', 'Income', 'Education', 'PolitSocial_1',␣
 ↪'PolitEcon_1', 'HowRelig_1', 'Fluency']
keep = cond + scores + attn + demos
s2r = s2r[keep]
s2r['Age'] = s2r['Age'].str.extract('(\d+)', expand = False)
num = scores + ['Check100_1', 'Check0_1', 'Age', 'Duration (in seconds)']
s2r[num] = s2r[num].apply(pd.to_numeric)
s2r[num] = s2r[num].apply(lambda x: x.fillna(x.mean()), axis = 0)
#Run exclusions
print('Pre-exclusions:\t' + str(len(s2r)))
s2r = s2r[s2r['Duration (in seconds)'] >= 240]
print('Post-duration exclusions:\t' + str(len(s2r)))
```

```
s2r = s2r[s2r['Age'] >= 18]
print('Post-age exclusions:\t' + str(len(s2r)))
s2r = s2r[s2r['Fluency'] != 'Not Fluent']
print('Post-fluency exclusions:\t' + str(len(s2r)))
s2r = s2r[(s2r['CheckNo1Wrong'].isnull()) | (s2r['CheckNo2Wrong'].isnull())]
#wrong = wrong[wrong['CheckNo2Wrong'].isnull()]
s2r = s2r[s2r['Check100_1'] >= 95]
s2r = s2r[s2r['Check0_1'] <= 5]
print('Post-attention checks:\t' + str(len(s2r)))
#Lowercase the botcheck and remove whitespace
s2r['BotCheckSET'] = s2r['BotCheckSET'].str.lower()
s2r['BotCheckSET'] = s2r['BotCheckSET'].str.strip()
s2r['BotCheckSET'] = s2r['BotCheckSET'].str.replace(" ","")
#Calculates distance and edits from the correct answer
s2r = s2r[s2r['BotCheckSET'].notna()]
s2r['bot_dist'] = s2r['BotCheckSET'].apply(lambda x: distance(x, 'set'))
s2r = s2r[(s2r['bot_dist'] <= 2) | (s2r['BotCheckSET'].str.contains('seta')) |
              (s2r['BotCheckSET'].str.contains('setb')) | (s2r['BotCheckSET'].
 ↪str.contains('setc'))]
print('Post-bot check:\t' + str(len(s2r)))
s2r.drop(columns = ['bot_dist'], inplace = True)
```

```
Pre-exclusions: 1822
Post-duration exclusions:       1749
Post-age exclusions:    1747
Post-fluency exclusions:        1735
Post-attention checks:  1373
Post-bot check: 1320
```

[53]:
```
print("Difference:\t\t{}".format(1822-1320))
```

```
Difference:             502
```

[54]:
```
s2r["Gender"].value_counts()
```

[54]:
```
Man      759
Woman    554
Other      6
Name: Gender, dtype: int64
```

[55]:
```
ageM = round(s2r["Age"].mean(), 2)
ageSD = round(s2r["Age"].std(), 2)
ageMin = s2r["Age"].min()
ageMax = s2r["Age"].max()
print("Ages {} to {}, Mean = {}, SD = {}".format(ageMin, ageMax, ageM, ageSD))
```

```
Ages 18.0 to 73.0, Mean = 35.33, SD = 10.58
```

[56]:
```
s2r['Age'] = round(s2r["Age"])
conditions = [
    s2r["Age"].isin(range(18,28)),
    s2r["Age"].isin(range(28,38)),
    s2r["Age"].isin(range(38,48)),
    s2r["Age"].isin(range(48,58)),
    s2r["Age"] >= 58,
    s2r["Age"].isna(),
]

choices = ["18-27", "28-37", "38-47", "48-57", "58+", "Missing"]

s2r["age_brackets"] = np.select(conditions, choices, default = 'what')
age = pd.DataFrame(s2r["age_brackets"].value_counts())
age["Sample%"] = round((age["age_brackets"]/len(s2r)) * 100, 2)
age
```

[56]:

	age_brackets	Sample%
28-37	562	42.58
18-27	329	24.92
38-47	230	17.42
48-57	135	10.23
58+	64	4.85

[57]:
```
s2r["Ethnicity"] = np.where(s2r["Ethnicity"].isna(), "Missing",␣
↪s2r["Ethnicity"])
race = pd.DataFrame(s2r["Ethnicity"].value_counts())
race["Sample%"] = round((race["Ethnicity"]/len(s2r)) * 100, 2)
race
```

[57]:

	Ethnicity	Sample%
White	930	70.45
Black/African American	175	13.26
Asian	92	6.97
Hispanic/Latinx	89	6.74
Other	19	1.44
American Indian/Alaska Native	12	0.91
Missing	2	0.15
Hawaiian/Pacific Islander	1	0.08

[58]:
```
s2r["Gender"] = np.where(s2r["Gender"].isna(), "Missing", s2r["Gender"])
gndr = pd.DataFrame(s2r["Gender"].value_counts())
gndr["Sample%"] = round((gndr["Gender"]/len(s2r)) * 100, 2)
gndr
```

[58]:

	Gender	Sample%
Man	759	57.50

```
Woman        554    41.97
Other          6     0.45
Missing        1     0.08
```

[59]:
```
s2_model_data = s2[s2['Gender'] != 'Other']
s2_model_data['Gender'] = np.where(s2_model_data['Gender'] == 'Man', 0, 1)

s2_model_data['Ethnicity'] = np.where(s2_model_data['Ethnicity'] == 'White', 0,␣
↪1)

lower = ['Under 5,000', '5,001 - 10,000', '10,001 - 15,000', '15,001 - 25,000',␣
↪'25,001 - 35,000']
s2_model_data['Income'] = np.where(s2_model_data['Income'].isin(lower), 0, 1)

m_econ = s2_model_data['PolitEcon_1'].mean()
m_soc = s2_model_data['PolitSocial_1'].mean()
m_relig = s2_model_data['HowRelig_1'].mean()
s2_model_data['poli_econ'] = np.where(s2_model_data['PolitEcon_1'] <= m_econ,␣
↪0, 1)
s2_model_data['poli_social'] = np.where(s2_model_data['PolitSocial_1'] <=␣
↪m_soc, 0, 1)
s2_model_data['how_religious'] = np.where(s2_model_data['HowRelig_1'] <=␣
↪m_relig, 0, 1)
s2_model_data = s2_model_data.dropna()
```

[60]:
```
fml = 'wrongness ~ C(Gender) + C(Income) + C(how_religious) + C(poli_social) +␣
↪C(poli_econ)'
vcf = {'relationship': '0 + C(relationship)'}
model = sm.MixedLM.from_formula(
    fml,
    vc_formula = vcf,
    groups = 'ResponseId',
    data = s2_model_data)
result = model.fit(method='powell')

print(result.summary())
var_resid = result.scale
var_random_effect = float(result.summary().tables[1].iloc[-1][0])
var_fixed_effect = result.predict(s2_model_data).var()
total_var = var_fixed_effect + var_random_effect + var_resid
marginal_r2 = var_fixed_effect / total_var
conditional_r2 = (var_fixed_effect + var_random_effect) / total_var
print('Conditional R2 = {}'.format(round(conditional_r2, 5)))
print("Converged:\t{}".format(result.converged))
```

```
             Mixed Linear Model Regression Results
================================================================
```

```
Model:               MixedLM   Dependent Variable:   wrongness
No. Observations:    5355      Method:               REML
No. Groups:          595       Scale:                1187.3293
Min. group size:     9         Log-Likelihood:       -26677.5757
Max. group size:     9         Converged:            Yes
Mean group size:     9.0
----------------------------------------------------------------
                      Coef.  Std.Err.   z    P>|z| [0.025 0.975]
----------------------------------------------------------------
Intercept             39.853    1.350 29.527 0.000 37.208 42.498
C(Gender)[T.1]        -0.116    1.222 -0.095 0.924 -2.510  2.279
C(Income)[T.1]         1.488    1.202  1.238 0.216 -0.868  3.845
C(how_religious)[T.1]  8.932    1.220  7.319 0.000  6.540 11.324
C(poli_social)[T.1]    1.055    1.549  0.681 0.496 -1.981  4.092
C(poli_econ)[T.1]     -0.484    1.540 -0.314 0.753 -3.502  2.534
relationship Var      74.404    0.370
================================================================

Conditional R2 = 0.07362
Converged:      True
```

[61]:

```python
functions = ['Care', 'Hierarchy', 'Mating', 'Reciprocity']
for i in range(0, len(functions)):
    cur_df = s2_model_data[s2_model_data['function'] == functions[i]]
    fml = 'wrongness ~ C(Gender) + C(Income) + C(how_religious) +␣
 ↪C(poli_social) + C(poli_econ)'
    vcf = {'relationship': '0 + C(relationship)'}
    model = sm.MixedLM.from_formula(
        fml,
        vc_formula = vcf,
        groups = 'ResponseId',
        data = cur_df)
    result = model.fit(method='powell')

    print('*'*75)
    print(functions[i])
    print('*'*75)
    print(result.summary())
    var_resid = result.scale
    var_random_effect = float(result.summary().tables[1].iloc[-1][0])
    var_fixed_effect = result.predict(cur_df).var()
    total_var = var_fixed_effect + var_random_effect + var_resid
    marginal_r2 = var_fixed_effect / total_var
    conditional_r2 = (var_fixed_effect + var_random_effect) / total_var
    print('Conditional R2 = {}'.format(round(conditional_r2, 5)))
    print("Converged:\t{}".format(result.converged))
```

```
***************************************************************************
```

```
Care
**************************************************************************
             Mixed Linear Model Regression Results
=================================================================
Model:               MixedLM    Dependent Variable:    wrongness
No. Observations:    897        Method:                REML
No. Groups:          299        Scale:                 427.2777
Min. group size:     3          Log-Likelihood:        -4141.1081
Max. group size:     3          Converged:             Yes
Mean group size:     3.0
-----------------------------------------------------------------
                       Coef.  Std.Err.   z    P>|z| [0.025 0.975]
-----------------------------------------------------------------
Intercept              57.202    2.730 20.955 0.000 51.852 62.552
C(Gender)[T.1]          3.510    2.489  1.410 0.158 -1.368  8.388
C(Income)[T.1]          0.242    2.433  0.100 0.921 -4.526  5.011
C(how_religious)[T.1]   3.516    2.450  1.435 0.151 -1.286  8.319
C(poli_social)[T.1]    -0.188    3.522 -0.053 0.957 -7.091  6.714
C(poli_econ)[T.1]       1.648    3.494  0.472 0.637 -5.201  8.496
relationship Var      287.239    2.096
=================================================================

Conditional R2 = 0.40755
Converged:      True
**************************************************************************
Hierarchy
**************************************************************************
             Mixed Linear Model Regression Results
==================================================================
Model:                MixedLM    Dependent Variable:    wrongness
No. Observations:     888        Method:                REML
No. Groups:           296        Scale:                 289.1218
Min. group size:      3          Log-Likelihood:        -4012.1862
Max. group size:      3          Converged:             Yes
Mean group size:      3.0
------------------------------------------------------------------
                       Coef.  Std.Err.   z    P>|z|  [0.025 0.975]
------------------------------------------------------------------
Intercept              53.762    3.070 17.510 0.000  47.744 59.780
C(Gender)[T.1]          1.511    2.766  0.546 0.585  -3.911  6.933
C(Income)[T.1]          0.753    2.735  0.276 0.783  -4.607  6.114
C(how_religious)[T.1]   8.443    2.805  3.010 0.003   2.946 13.940
C(poli_social)[T.1]    -1.337    3.239 -0.413 0.680  -7.687  5.012
C(poli_econ)[T.1]      -3.778    3.229 -1.170 0.242 -10.106  2.550
relationship Var      425.735    3.113
==================================================================

Conditional R2 = 0.6095
```

```
Converged:      True
**************************************************************************
Mating
**************************************************************************
              Mixed Linear Model Regression Results
=================================================================
Model:              MixedLM   Dependent Variable:    wrongness
No. Observations:   1785      Method:                REML
No. Groups:         595       Scale:                 260.6134
Min. group size:    3         Log-Likelihood:        -7926.0707
Max. group size:    3         Converged:             Yes
Mean group size:    3.0
-----------------------------------------------------------------
                       Coef.  Std.Err.   z    P>|z| [0.025 0.975]
-----------------------------------------------------------------
Intercept              -0.072    1.832 -0.039 0.969 -3.663  3.519
C(Gender)[T.1]         -2.936    1.659 -1.770 0.077 -6.187  0.314
C(Income)[T.1]          5.092    1.632  3.119 0.002  1.892  8.291
C(how_religious)[T.1]  16.531    1.657  9.979 0.000 13.284 19.778
C(poli_social)[T.1]     3.173    2.103  1.509 0.131 -0.949  7.296
C(poli_econ)[T.1]       2.003    2.090  0.958 0.338 -2.094  6.099
relationship Var      293.392    1.678
=================================================================

Conditional R2 = 0.58635
Converged:      True
**************************************************************************
Reciprocity
**************************************************************************
             Mixed Linear Model Regression Results
================================================================
Model:               MixedLM   Dependent Variable:   wrongness
No. Observations:    1785      Method:               REML
No. Groups:          595       Scale:                753.0728
Min. group size:     3         Log-Likelihood:       -8487.6526
Max. group size:     3         Converged:            Yes
Mean group size:     3.0
----------------------------------------------------------------
                      Coef.  Std.Err.   z    P>|z| [0.025 0.975]
----------------------------------------------------------------
Intercept             64.267    1.628 39.486 0.000 61.077 67.457
C(Gender)[T.1]         0.263    1.473  0.178 0.858 -2.625  3.150
C(Income)[T.1]        -0.949    1.450 -0.654 0.513 -3.791  1.893
C(how_religious)[T.1]  4.013    1.472  2.727 0.006  1.129  6.898
C(poli_social)[T.1]    0.201    1.868  0.108 0.914 -3.460  3.863
C(poli_econ)[T.1]     -2.037    1.857 -1.097 0.273 -5.676  1.602
relationship Var      49.010    0.779
================================================================
```

```
Conditional R2 = 0.06705
Converged:       True
```

2.3 Stage 3

3.1

[62]:
```
s3r = pd.read_csv('sample3_raw.csv', lineterminator = '\n')
pre_excl = s3r.copy()
print('Pre-exclusions:\t\t', len(s3r))
s3r = s3r[(s3r['Att100_1'].notnull()) & (s3r['Att0_1'].notnull()) &␣
↪(s3r['Att100b_1'].notnull())]
s3r[['Att100_1', 'Att0_1', 'Att100b_1']] = s3r[['Att100_1', 'Att0_1',␣
↪'Att100b_1']].astype(int)
s3r = s3r[(s3r['Att100_1'] >= 95) & (s3r['Att0_1'] <= 5) & (s3r['Att100b_1'] >=␣
↪95)]
print('Post-exclusion:\t\t', len(s3r))
```

```
Pre-exclusions:          149
Post-exclusion:          85
```

[63]:
```
print("Difference:\t\t{}".format(149-85))
```

```
Difference:              64
```

[64]:
```
s3r['Q125'].value_counts()
```

[64]:
```
Male          46
Female        38
Nonbinary      1
Name: Q125, dtype: int64
```

[65]:
```
ageM = round(s3r["Q126"].mean(), 2)
ageSD = round(s3r["Q126"].std(), 2)
ageMin = s3r["Q126"].min()
ageMax = s3r["Q126"].max()
print("Ages {} to {}, Mean = {}, SD = {}".format(ageMin, ageMax, ageM, ageSD))
```

```
Ages 21 to 69, Mean = 34.82, SD = 10.66
```

[66]:
```
conditions = [
    s3r["Q126"].isin(range(18,28)),
    s3r["Q126"].isin(range(28,38)),
    s3r["Q126"].isin(range(38,48)),
    s3r["Q126"].isin(range(48,58)),
    s3r["Q126"] >= 58,
    s3r["Q126"].isna(),
```

```
]

choices = ["18-27", "28-37", "38-47", "48-57", "58+", "Missing"]

s3r["age_brackets"] = np.select(conditions, choices)
age = pd.DataFrame(s3r["age_brackets"].value_counts())
age["Sample%"] = round((age["age_brackets"]/len(s3r)) * 100, 2)
age
```

[66]:

	age_brackets	Sample%
28-37	37	43.53
18-27	23	27.06
38-47	14	16.47
48-57	6	7.06
58+	5	5.88

[67]:

```
s3r["Q127"] = np.where(s3r["Q127"] == 'Black/African,Caucasian/White',␣
↪'Multiracial', s3r["Q127"])
s3r["Q127"] = np.where(s3r["Q127"] == 'Asian,Caucasian/White', 'Multiracial',␣
↪s3r["Q127"])
s3r["Q127"] = np.where(s3r["Q127"].isna(), "Missing", s3r["Q127"])
race = pd.DataFrame(s3r["Q127"].value_counts())
race["Sample%"] = round((race["Q127"]/len(s3r)) * 100, 2)
race
```

[67]:

	Q127	Sample%
Caucasian/White	59	69.41
Black/African	12	14.12
Asian	5	5.88
Hispanic/Latinx	4	4.71
Multiracial	3	3.53
Native American	2	2.35

[68]:

```
s3r["Q125"] = np.where(s3r["Q125"].isna(), "Missing", s3r["Q125"])
gndr = pd.DataFrame(s3r["Q125"].value_counts())
gndr["Sample%"] = round((gndr["Q125"]/len(s3r)) * 100, 2)
gndr
```

[68]:

	Q125	Sample%
Male	46	54.12
Female	38	44.71
Nonbinary	1	1.18
